# Arbovirus diagnostics: from conventional methods to emerging technologies—a chikungunya virus-centric review

**DOI:** 10.3389/fmicb.2026.1960039

**Published:** 2026-10-06

**Authors:** Yongqing Zhu, Jiaxuan Li, Haiyan Mao, Keda Chen, Jianhua Li

**Affiliations:** 1 Zhejiang Key Laboratory of Public Health Detection and Pathogenesis Research, Department of Microbiology, Zhejiang Provincial Center for Disease Control Prevention, Hangzhou, China; 2 New Medical Science Center of Anhui Medical University, Hefei, China; 3 Key Laboratory of Artificial Organs and Computational Medicine in Zhejiang Province, Shulan International Medical College, Zhejiang Shuren University, Hangzhou, China

**Keywords:** arboviruses, chikungunya virus, diagnostic techniques, emerging technologies, point-of-care testing

## Abstract

Arboviruses remain an important global health concern because of their expanding distribution, diverse transmission patterns, and the challenges associated with accurate and timely diagnosis. This review is centered on Chikungunya virus (CHIKV) as a representative model, focusing on diagnostic strategies across different stages of infection and the key diagnostic pitfalls that affect CHIKV detection in co-circulation settings. In this review, we summarize current laboratory-based approaches for CHIKV detection and differential diagnosis, including virus isolation, antigen detection, nucleic acid amplification and serological assays, with discussion of their applications, strengths and limitations. We further review recent developments in emerging diagnostic technologies, including next-generation sequencing, CRISPR-based detection methods, aptamer-based recognition systems, biosensors, and point-of-care testing platforms, highlighting their potential advantages and current limitations. Comparative examples involving dengue virus (DENV) and Zika virus (ZIKV) are discussed specifically to illustrate cross-reactivity and misclassification risks relevant to CHIKV diagnosis. Particular attention is given to persistent issues affecting diagnostic accuracy and practical implementation, including serological cross-reactivity among co-circulating arboviruses and the challenges of translating advanced technologies into accessible tools for field and resource-limited settings. Overall, this review summarizes progress in CHIKV-relevant diagnostics and outlines future opportunities for developing more reliable, rapid, and widely applicable detection strategies.

## 1 Introduction

Arboviruses (arthropod-borne viruses) comprise a taxonomically diverse group of viruses transmitted through the bites of hematophagous arthropods (such as Aedes, Culex, Ticks), posing a substantial and growing threat to global public health and animal populations (Siew et al., 2025). These pathogens are maintained in nature through an enzootic maintenance cycle, where wild animals-particularly birds and certain mammals-may act as primary reservoir or amplifying hosts. Human and other susceptible vertebrate infections occur when infected arthropod vectors transfer the virus during blood-feeding, leading to spillover events from reservoir species to accidental hosts (Power et al., 2022). Recent detection of dengue virus serotype 2 in Malaysian treeshrew feces further illustrates the value of investigating diverse wildlife species in arbovirus surveillance; however, their role in Chikungunya virus (CHIKV) transmission requires specific evidence (Siew et al., 2026). In medicine, human to human transmission is mainly mediated by mosquitoes, ticks and so on (Varghese et al., 2023). Structurally, the genomes of arboviruses may consist of positive- or negative-sense single-stranded RNA, and some arboviruses have segmented genomes. In many medically important arboviruses, the genomic RNA is associated with capsid or nucleoprotein proteins and enclosed by a host-derived lipid bilayer studded with virally encoded glycoproteins. These glycoproteins mediate critical early infection steps, including attachment to host-cell surface factors or receptors and membrane fusion (Hernandez et al., 2014; Holmes et al., 2020; Liu et al., 2024). Of the arbovirus families associated with human disease, *Flaviviridae, Togaviridae*, and bunyavirus-related families are particularly important. Notable human pathogens include Zika virus (ZIKV), dengue virus (DENV), and West Nile virus (WNV) of the family Flaviviridae, as well as CHIKV, an alphavirus of the family *Togaviridae*.

In recent years, CHIKV has transitioned from a geographically restricted disease to a global public health concern (Wang et al., 2026; Zhang et al., 2026). According to surveys, 119 countries worldwide have reported CHIKV outbreaks, with 104 countries experiencing local virus transmission and an estimated 2.8 billion people at risk. The epidemiological patterns vary across regions, which can be broadly categorized into two types: endemic and outbreak epidemic (Kang et al., 2024; Ribeiro dos Santos et al., 2025). The local epidemic persists in a specific population group within a certain area and has a relatively stable incidence rate (Umaña et al., 2026). A sudden epidemic occurs when the incidence rate within a specific area exceeds the usual level of previous years, indicating a change in the pathogen's mutation or transmission pattern, or a weakening of the population's immune barrier (Thompson et al., 2026). The Americas bear the heaviest disease burden. In 2024, the incidence rate in the Americas reached as high as 43.9 cases per 100,000 population, with over 430,000 reported cases. Southeast Asia is another major endemic region, with an incidence rate of 14.3 cases per 100,000 population in 2024 (Wang et al., 2026). The outbreak has also spread to areas that previously only reported imported cases. In 2025, China's Guangdong Province experienced the largest outbreak on record, reporting thousands of locally acquired cases within a short period, with subsequent spread to regions such as Macau and Hong Kong (Tee et al., 2025; Dai et al., 2026). In endemic tropical and subtropical regions, the early clinical manifestations of infections caused by mosquito-borne viruses—namely CHIKV, DENV, and ZIKV—are highly similar. During the acute phase of infection, patients typically present with sudden onset of high fever, headache, myalgia, arthralgia, and rash (Carrillo et al., 2025), this makes it extremely difficult to distinguish the infecting virus based solely on clinical presentation, and also increases the risk of misdiagnosis and underdiagnosis (Ferdous et al., 2026). Moreover, the epidemiological profiles of these viruses predispose individuals to co-infection with multiple viruses, further complicating diagnosis (Aurelio et al., 2026).

Collectively responsible for an estimated 400 million annual infections globally, these viruses pose a significant clinical burden due to complications such as neuroinvasive encephalitis and chronic arthritis. The spectrum of resultant human disease ranges from mild, self-limiting febrile illness to severe, life-threatening conditions (Liu et al., 2024; Wang et al., 2025). The global spread of arboviral diseases is being accelerated by a complex convergence of environmental and socioeconomic drivers. Climate change, landscape transformation, and rapid urban expansion are creating ideal ecological conditions for mosquito population growth and viral transmission. Meanwhile, expanding international trade and persistent economic disparities between regions are facilitating the movement of both insect vectors and viruses across borders. These interconnected factors collectively contribute to the rising global incidence and expanding geographical reach of arboviral infections (Power et al., 2022; Jibowu et al., 2025).

The significant public health burden posed by arboviruses underscores the need for diagnostic tools that are highly sensitive, rapid, and accurate-capabilities essential for delivering appropriate clinical management, enabling effective outbreak control, and supporting comprehensive epidemiological surveillance. Standard arbovirus diagnostic approaches include serological testing, molecular analysis, and viral culture techniques. The diagnosis of viral infections via serological methods relies on detecting specific antibodies (IgM and IgG) in the blood. Two widely adopted techniques are the enzyme-linked immunosorbent assay (ELISA), used to identify binding antibodies, and the virus neutralization test (VNT), which confirms the presence of neutralizing antibodies. While valuable for large-scale screening, both methods are hampered by serological cross-reactivity among related viruses. Moreover, their accuracy heavily depends on sample collection timing relative to symptom onset (Allen et al., 2020). Studies indicate that dried serum spots offer a practical solution for sample collection and transport. However, whether it performs as well as conventional liquid serum in diagnostic tests still needs proper validation (Würsch et al., 2023). Reverse transcription PCR-based methods, particularly real-time reverse transcription quantitative PCR(RT-qPCR), are widely used for arbovirus detection. These methods enable the specific detection of viral RNA and can provide high analytical sensitivity and specificity. RT-qPCR is widely used for detecting acute arboviral infections, although its implementation in large-scale screening programs remains constrained by substantial operational costs (Rutkowski et al., 2020; Kasbergen et al., 2025; Louis et al., 2025). Viral culture, while invaluable for virus isolation and characterization, demands lengthy procedures and specialized biosafety facilities, making it less suitable for rapid diagnostic deployment in outbreak settings. Based on the existing technological framework, the core difficulties in CHIKV-relevant arboviral diagnosis lie in two aspects: the unavoidable window period of serological testing, and the antibody cross-reactivity arising from antigenic homology among closely related arboviruses. These limitations are particularly consequential for CHIKV diagnosis in settings where other co-circulating arboviruses share overlapping early clinical features and serological targets.

We focus on CHIKV as the central example because its diagnosis is particularly sensitive to (1) a narrow early nucleic-acid window driven by rapid viral-load decline and (2) frequent clinical overlap with other co-circulating arboviruses, which complicates interpretation of both molecular and serological results. Accordingly, diagnostic approaches discussed throughout this review are explicitly framed around CHIKV diagnostic decision points and common sources of misclassification. As summarized in Figure 1, CHIKV-relevant diagnostic approaches can be broadly categorized into three groups, covering testing during the acute and convalescent phases of infection, with specific measures implemented to minimize cross-reactivity. Although traditional viral detection techniques continue to play essential roles in clinical practice, a new generation of innovative technologies is actively reshaping the CHIKV diagnostic landscape. Leveraging national policy initiatives is crucial for driving the technological innovation required to strengthen our response to arboviral diseases and safeguard global health security.

**Figure 1:**
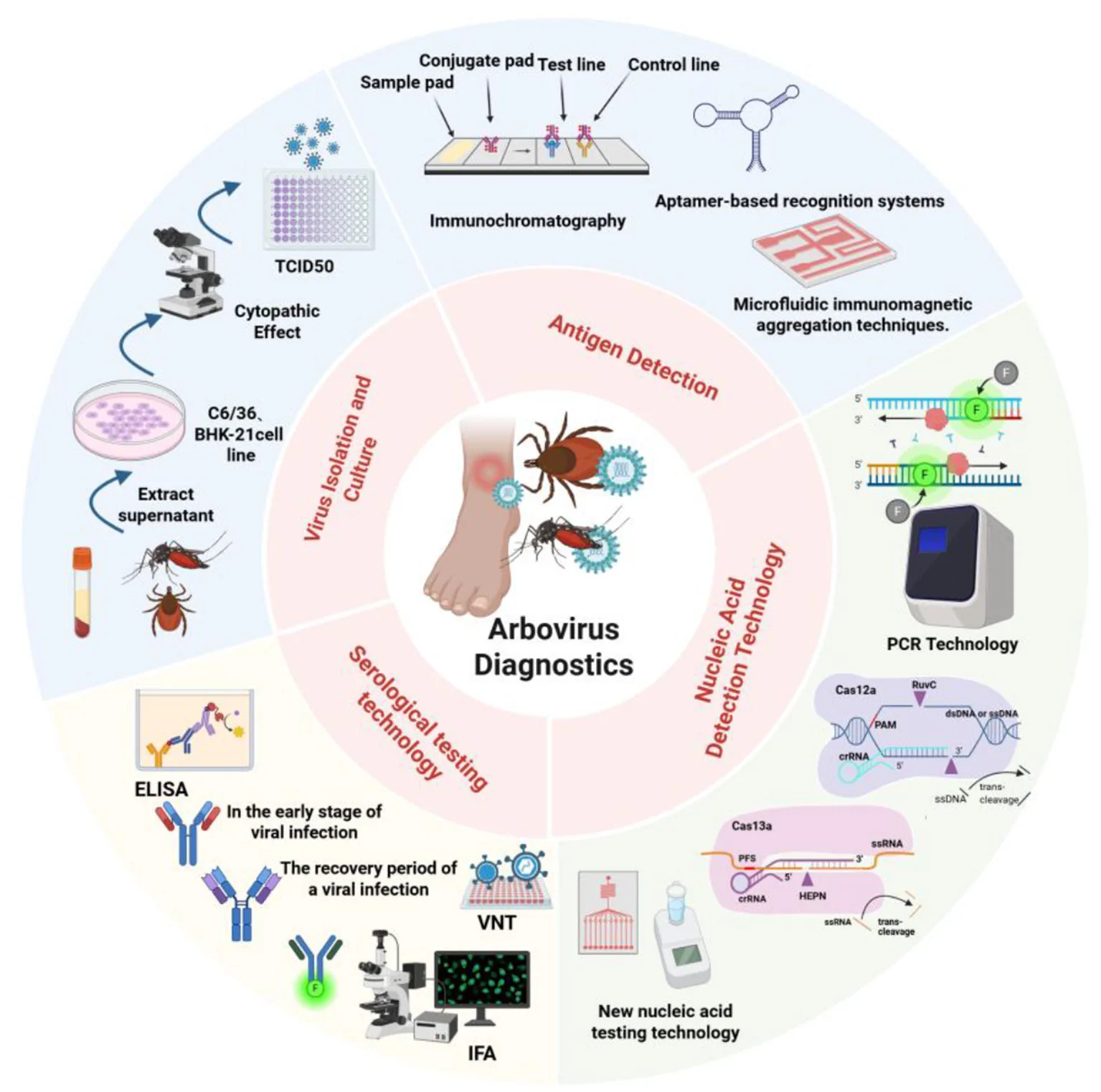
Schematic overview of diagnostic strategies for CHIKV-relevant arbovirus. Conventional methods include virus isolation and antigen detection. Molecular methods comprise PCR-based and novel nucleic acid detection techniques, including CRISPR-Cas and isothermal amplification. Serological approaches include ELISA, Immunofluorescence assays (IFA), and Virus neutralization test (VNT), with specific applications in acute and convalescent phases and measures to reduce cross-reactivity.

## 2 Literature search and selection

A literature search was conducted using PubMed to identify relevant studies on arbovirus diagnostics, with particular emphasis on chikungunya virus (CHIKV). The retrieved records were screened according to their relevance to diagnostic methodologies, analytical performance, cross-reactivity, point-of-care testing, and emerging diagnostic technologies. After screening and eligibility assessment, 164 publications were selected for inclusion in this review. The overall literature identification and selection process is summarized in Figure 2.

**Figure 2:**
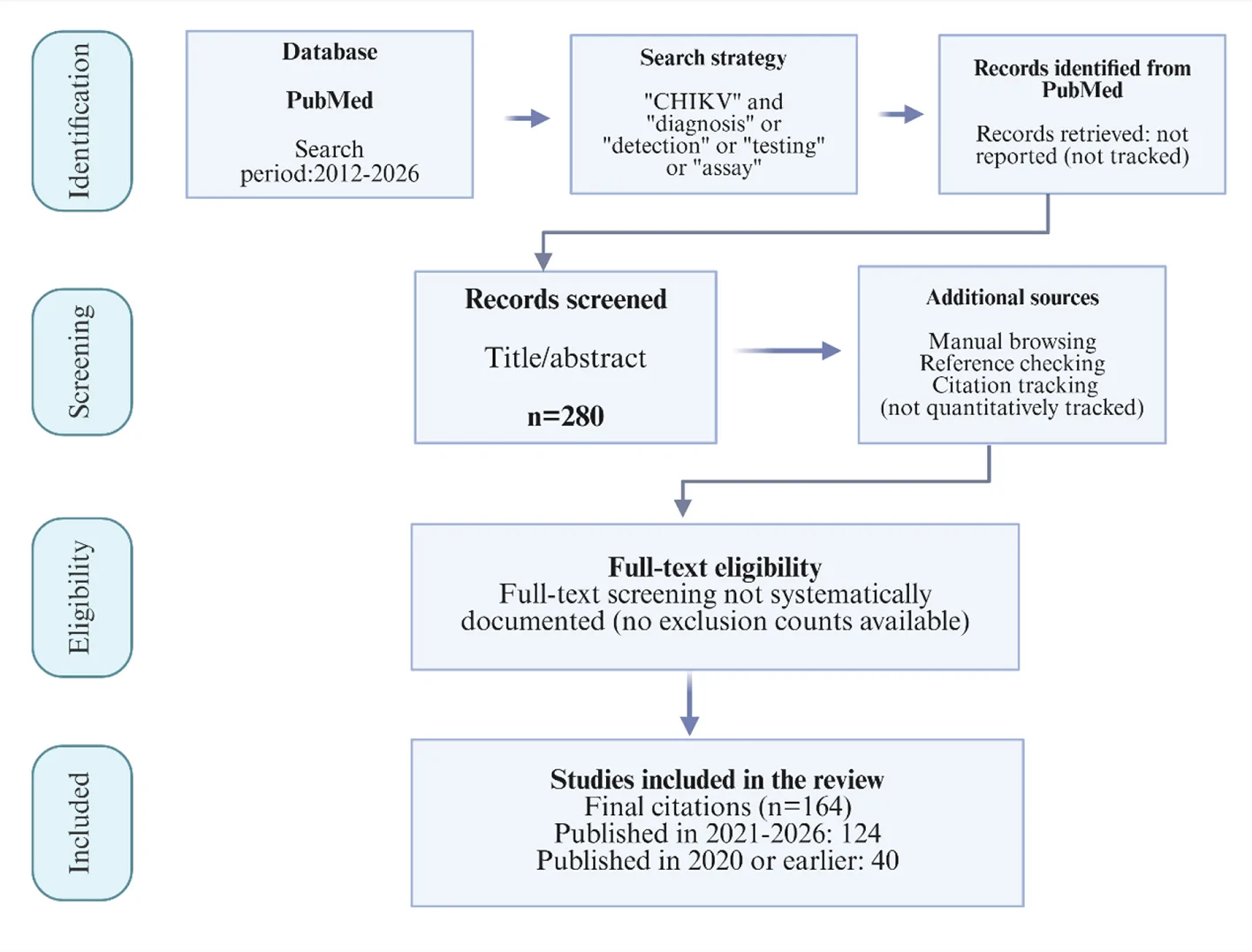
PRISMA flow diagram illustrating the identification, screening, eligibility assessment, and inclusion of publications in this review.

## 3 Diagnostic windows and virological insights

### 3.1 Chikungunya virus: from viral biology to diagnostic challenges

CHIKV is a mosquito-borne, single-stranded positive-sense RNA virus belonging to the genus *Alphavirus* within the family *Togaviridae*. It is primarily transmitted by *Aedes aegypti* and *Aedes albopictus*, which have facilitated its rapid geographic expansion in tropical and subtropical regions. CHIKV was first isolated in Tanzania in 1952 and was named “Chikungunya,” meaning “to bend up,” referring to the characteristic stooped posture caused by severe arthralgia experienced by infected patients (Simo et al., 2019; de Lima Cavalcanti et al., 2022; Freppel et al., 2024). Based on phylogenetic characteristics, CHIKV is classified into three major genotypes, including the West African, Asian, and East/Central/South African (ECSA) lineages, all of which have contributed to global outbreaks (Badar et al., 2021). The distribution and transmission dynamics of CHIKV are closely associated with mosquito vector expansion, climate conditions, and human mobility. Currently, CHIKV represents a significant global public health concern, with an estimated 16.9 million infections occurring annually and approximately 2.8 billion people living in areas at risk of exposure (de Souza et al., 2025). Therefore, the increasing global burden of CHIKV infection highlights the urgent need for rapid and reliable diagnostic strategies.

Early and accurate diagnosis of CHIKV remains challenging due to the dynamic changes in viral load and the limited diagnostic window during infection the specific content can be seen in Figure 3C. In general, CHIKV infection is associated with relatively high viremia during the first week after symptom onset, which represents the principal window for nucleic acid detection. Although viral load may vary and some early specimens can still exhibit low viral RNA levels. Thereby increasing the risk of false-negative RT-qPCR results. False negatives are more often driven by inappropriate sampling time, declining viremia, suboptimal specimen quality, RNA degradation, and sequence variation (Johnson et al., 2016; Broeders et al., 2020; Andrew et al., 2023; Khongwichit et al., 2025). Viral RNA is most readily detectable within the first week after symptom onset. Reported serum detection rates vary across cohorts and assay formats and can reach high levels in some studies. Viral loads rapidly decline after the viremic phase and the period of detectable viremia is typically limited reported durations vary, roughly over several days to about a week (Martins et al., 2022; Sajith et al., 2025). In addition, the clinical manifestations of CHIKV infection, including fever, arthralgia, and rash, overlap substantially with other mosquito-borne viral diseases such as DENV and ZIKV, increasing the possibility of misdiagnosis and highlighting the need for more sensitive and complementary diagnostic approaches (Cherabuddi et al., 2016; Jiang et al., 2024; Polanía-Espinosa et al., 2025).

**Figure 3:**
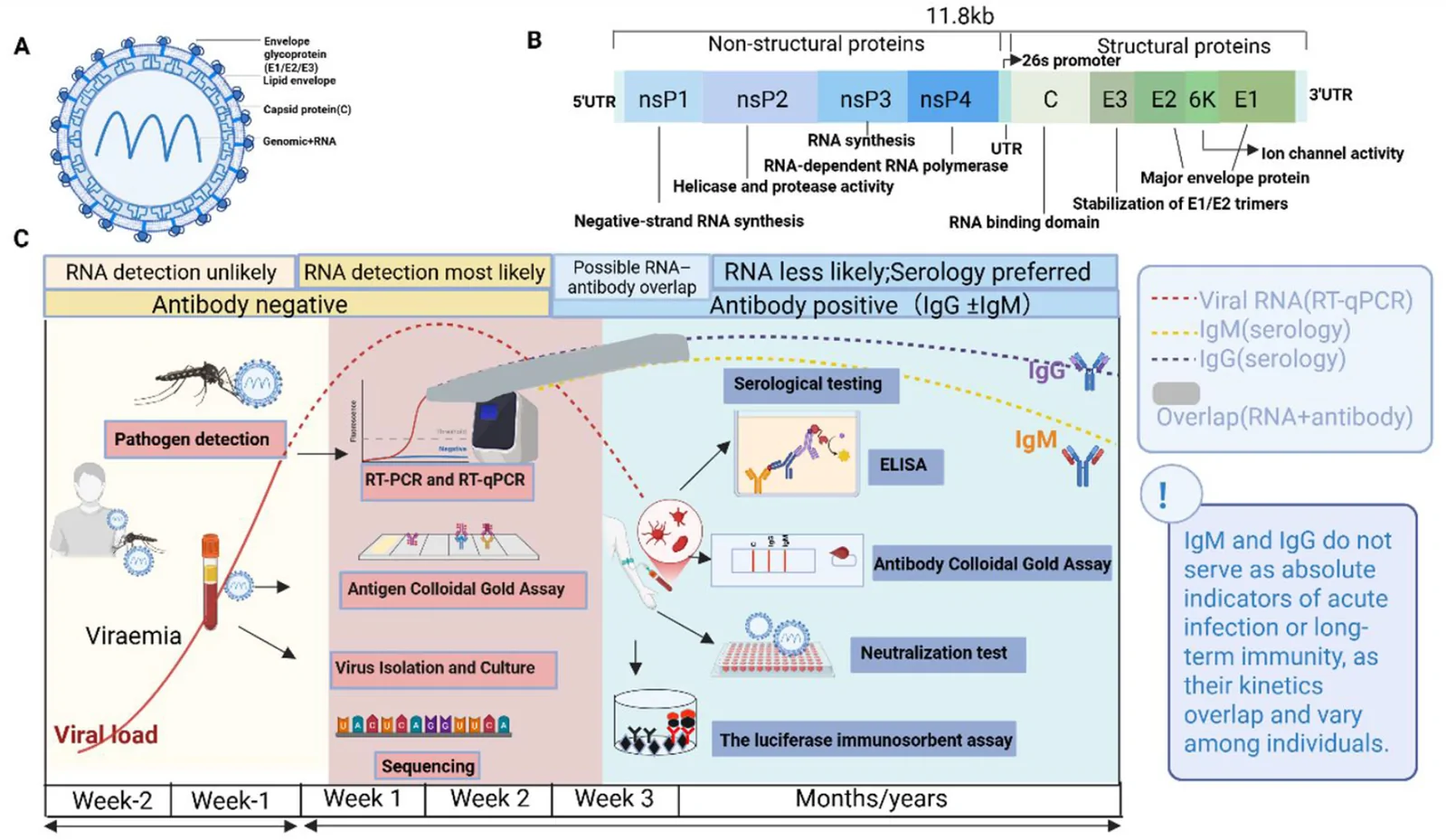
Structure, genome, and immune/diagnostic dynamics of Chikungunya virus (CHIKV). **(A)** Schematic of the CHIKV virion, showing the capsid-encapsulated positive-sense RNA genome, host-derived lipid envelope, and surface envelope glycoprotein (E1/E2/E3) complexes. **(B)** CHIKV genomic organization (about 11.8 kb), with annotated non-structural (nsP1-nsP4, replication-associated) and structural (C, E3, E2, 6K, E1, virion assembly/entry) protein coding regions, and key functional activities. **(C)** Schematic temporal dynamics of viremia, viral RNA detection, CHIKV-specific IgM and IgG responses, and corresponding diagnostic approaches relative to symptom onset. Viral RNA is generally most readily detected during the early viremic phase, when RT-PCR/RT-qPCR, antigen-based assays, virus isolation, and sequencing may be informative. During seroconversion, viral RNA and antibody detection may overlap. Thereafter, serum RNA detection becomes less likely, whereas serological testing, including ELISA, rapid antibody assays, neutralization tests, and luciferase immunosorbent assays, becomes increasingly informative. The time windows shown are illustrative and may vary with individual immune kinetics, specimen type, viral load, assay sensitivity, and sampling time. IgM and IgG responses may overlap and should not be interpreted as absolute indicators of acute infection or long-term protective immunity.

The CHIKV genome is approximately 11.8 kb in length and encodes four non-structural proteins (nsP1–nsP4) and five structural proteins, including capsid (C), envelope proteins E3, E2, 6K, and E1. The 6K gene produces two protein products, 6K and TF, via programmed-1 ribosomal frameshifting (Basore et al., 2019; Thannickal et al., 2024; de Souza et al., 2025). An overview of the viral structure and genome organization is presented in Figures 3A, B. Among these viral components, the envelope glycoproteins E1 and E2 have attracted considerable attention as diagnostic targets due to their functional importance and antigenic properties. The E2 protein is exposed on the surface of viral particles and mediates initial attachment to host cell receptors. Its high immunogenicity enables the induction of neutralizing antibodies, making it a key target for serological detection. Moreover, E2 exhibits relatively high conservation among different CHIKV genotypes, reducing the risk of detection failure caused by genetic variation and supporting its application as a diagnostic target (Rodrigues et al., 2020; Amaral et al., 2021; Powers et al., 2023; Goto et al., 2025). Following E2-mediated receptor binding, E1 facilitates membrane fusion and viral genome release into host cells. Conserved functional domains within E1, particularly the fusion peptide region, provide potential targets for broad-spectrum molecular detection strategies (Hoornweg et al., 2016; Mangala Prasad et al., 2022; Thannickal et al., 2024). Collectively, the biological characteristics of E1 and E2 make them important targets for the development of CHIKV diagnostic assays (Verma et al., 2022).

### 3.2 Diagnostic window

The diagnostic window for CHIKV is of critical importance for both clinical diagnosis and epidemiological surveillance. The diagnostic window refers to the period between viral infection and the point at which clinical laboratory tests can reliably detect the virus or its biomarkers. Two main types of detection methods are typically employed during this window: direct pathogen detection and serological testing (Andrew et al., 2022). Nucleic acid detection determines the presence of CHIKV in a sample by directly detecting viral RNA, thereby confirming infection. Nucleic acid-based techniques, particularly real-time reverse transcription quantitative polymerase chain reaction (RT-PCR), exhibit analytical sensitivity and specificity and have become a crucial tool for the early detection of CHIKV (Wang et al., 2016; Afzal et al., 2025; Sajith et al., 2025). Serological testing diagnoses CHIKV infection by detecting virus-specific antibodies, such as IgM and IgG, produced by the patient. Compared with direct pathogen detection, serological assays provide a longer diagnostic window. IgM antibodies typically appear 5–7 days post-infection and can persist for several weeks to months, whereas IgG antibodies arise 1–2 weeks after infection and may persist for years. Consequently, serological testing is particularly suitable for diagnosing infections during the later or convalescent phase (Ohst et al., 2018). The diagnostic window is critical for disease treatment, patient management, and the collection of epidemiological data. Due to the structural similarities between CHIKV and other arboviruses, laboratory testing during this window period is essential for accurate diagnosis.

### 3.3 Current detection methods and challenges

#### 3.3.1 Virus isolation and culture

Effective isolation of arboviruses relies on systematically acquiring specimens such as blood, tissues, or bodily fluids from hosts or vectors suspected of infection. The samples are homogenized by grinding and clarified by filtration to remove debris, obtaining a final homogenate suitable for inoculation, as shown in Figure 1 (virus isolation and culture). The resulting homogenate is then subjected to viral culture. The successful cultivation of arboviruses depends critically on the use of permissive cell lines that enable robust viral replication and amplification. Two widely adopted systems for this purpose are the C6/36 cell line (derived from *Aedes albopictus* larvae) and the BHK-21 line (established from Syrian hamster kidney tissue). These cell lines demonstrate pronounced susceptibility to diverse arboviruses, a trait attributable to intrinsic genomic features that promote efficient viral entry and sustained replication. After introduction into permissive cells, the virus first attaches to and enters through specific surface receptors, then releases its RNA to redirect the host's cellular machinery for replication (Miller et al., 2018; Fan et al., 2020). The viral titer is determined by a plaque assay, a method based on the principle of localized infection. Following the addition of virus to a susceptible cell monolayer, a semi-solid overlay restricts infection to adjacent cells. Each infectious virus particle then produces a distinct plaque-a visible zone of cell lysis-through repeated cycles of infection and cell death. The number of these plaques is directly proportional to the concentration of infectious virus in the original sample (Carneiro et al., 2022; Takumi-Tanimukai et al., 2022; Waturangi, 2024). The plaque assay enables direct measurement of replication-competent infectious virus and accurate viral titration, making it a widely used reference method for infectious virus quantification. However, the assay places stringent demands on both technical execution and cell culture conditions. Its performance depends critically on the quality and confluency of the host-cell monolayer, as variations in cell condition and density can affect plaque formation and compromise the reproducibility and reliability of viral titration (Shurtleff et al., 2012; Baer and Kehn-Hall, 2014; Mendoza et al., 2020).

#### 3.3.2 Antigen detection

Antigen detection techniques rely on highly specific antibodies to recognize viral proteins, including structural and non-structural components. Detection is subsequently achieved through molecular approaches such as enzyme-mediated colorimetric reactions or fluorescence-based assays. Within the laboratory diagnostic framework for arboviruses, antigen detection has emerged as an important complement to nucleic acid testing, owing to its operational simplicity, rapid turnaround time, and suitability for point-of-care and field applications (Alejo-Cancho et al., 2020). For infections caused by various viruses transmitted by Aedes mosquitoes, such as DENV, ZIKV, and CHIKV, antigen detection is particularly suitable for early screening during the acute phase (Sekaran et al., 2022). However, for CHIKV, antigen detection remains constrained by the extent of validation across settings and by limited uptake of standardized assays in routine clinical practice. As seen from the structural modules of the aforementioned viruses (Figure 3B), this open reading frames (ORF) is translated and cleaved into structural proteins. As mentioned, CHIKV belongs to the Alphavirus genus. Its genome is a single-stranded positive-sense RNA, which encodes two major open reading frames (ORF) for structural proteins (C, E3, E2, 6K, E1) and non-structural proteins (nsP1–nsP4) (Nunes et al., 2023; Wang et al., 2024). Among them, the envelope glycoproteins E1 and E2 are the main immunogenic antigens, capable of inducing the production of neutralizing antibodies in the host (Hamrahjoo et al., 2026). However, the expression of these antigens during the viral infection process shows a clear time-dependent pattern: the viremia of CHIKV typically lasts for a short duration (usually declining rapidly within a few days post-infection), resulting in a narrow window for detecting the antigens in bodily fluids. This, in turn, may limit the clinical sensitivity of antigen detection (Choudhary et al., 2021). Additionally, the E1/E2 proteins may exist in different conformations within the body, with the extent of epitope exposure being influenced by the viral maturation state and the host environment. This variability further complicates the consistency of antibody recognition (Cho et al., 2024; Thannickal et al., 2024). In addition, this article also highlights the potential issue of “cross-reactivity-like” interference in antigen detection. Despite differences in their viral families and antigenic structures, CHIKV, DENV, and ZIKV continue to present substantial challenges for differential diagnosis. Similar clinical symptoms, overlapping transmission patterns, and serological cross-reactivity among arboviruses can interfere with accurate identification, highlighting the need for highly specific diagnostic approaches (Aranda-Coello et al., 2025).

#### 3.3.3 Serological testing technology

##### 3.3.3.1 ELISA and IFA

Enzyme-linked immunosorbent assay (ELISA) and immunofluorescence assay (IFA) are widely used methods for the detection of IgG and IgM antibodies. IgM is the earliest antibody produced during the primary immune response and typically appears in the early stage of infection, serving as an indicator of acute infection (Shete et al., 2022; Zajac et al., 2023). IgG antibodies are produced at later stages of the immune response and are considered indicators of past exposure or secondary infection (Marfía et al., 2025). Enzyme-linked immunosorbent assay (ELISA) is based on the specific interaction between antigens and antibodies. By using enzyme-labeled antigens or antibodies, ELISA generates a detectable signal, such as a colorimetric change, through enzyme-catalyzed substrate reactions, allowing qualitative or quantitative detection of target antigens or antibodies. Depending on the detection format, ELISA can be classified into direct, indirect, sandwich, and competitive assays. Despite the widespread use of the aforementioned methods in clinical detection of arboviruses, serological testing for CHIKV still faces multiple challenges. Firstly, antibody production exhibits significant time-dependence, with variability in the timing and duration of IgM antibody appearance across different individuals (Ou et al., 2025). Furthermore, the long-term presence of IgG antibodies complicates the differentiation between recent infections and past exposures. Secondly, in regions where multiple arboviruses co-circulate, complex immune backgrounds (such as previous infections or co-infections) may interfere with the test results (Sena et al., 2024). As mentioned above, CHIKV, Dengue virus, and Zika virus belong to different virus families, and their antigenic structures differ significantly. However, in practical detection systems, cross-reactivity may still occur due to non-specific antibody binding or limitations in reagent design, leading to the aforementioned cross-reactivity issues (Aranda-Coello et al., 2025). In addition, among other pathogens within the Alphavirus genus, the structural homology of envelope proteins further exacerbates antibody cross-reactivity, which in turn may reduce the specificity of serological detection (Hatami et al., 2026). These factors collectively limit the accuracy of CHIKV serological testing and suggest that, in actual diagnostics, it is necessary to combine nucleic acid testing or neutralization assays to enhance the reliability of the results and improve their clinical interpretability.

### 3.4 Advances in CHIKV immunoassays

Beyond conventional serological assays, recent studies have focused on improving CHIKV immunodetection performance through optimized antigen selection and signal amplification strategies. Specific antigen detection targeting the E1 and E2 envelope proteins of CHIKV has been developed. These structural proteins, illustrated in Figure 3B, serve as primary targets for immunoassays. Recent studies have employed immunochromatographic rapid tests using mouse-derived monoclonal antibodies against the CHIKV E1 protein to detect multiple CHIKV genotypes, including the East/Central/South African and Asian genotypes, demonstrating high sensitivity and specificity in the reported evaluation (Okabayashi et al., 2015). More recently, recombinant antigen-based ELISA approaches have been developed, in which recombinant CHIKV E2 protein is used as a capture antigen to improve the sensitivity and specificity of anti-CHIKV antibody detection (Fumagalli et al., 2018). Furthermore, to enhance serological detection capability, multiplex E antigen-based immunoassays have been developed. By combining two or more recombinant CHIKV E antigens, these assays improve the recognition of anti-CHIKV IgG antibodies in patient sera. A recent study demonstrated that the combination of multiple E2 antigens achieved a 100% detection rate for anti-CHIKV IgG antibodies, with antibodies remaining detectable even 235 days after infection, highlighting its potential for long-term serological surveillance (Li et al., 2022). In contrast to RT-qPCR, which is mainly applicable during the early viremic phase, antibody-based assays provide advantages for retrospective diagnosis and epidemiological investigations. However, serological methods may still face challenges associated with antibody cross-reactivity among closely related arboviruses (Kasbergen et al., 2023). Fluorescence-linked immunosorbent assay (FLISA) has also been explored as a sensitive immunodetection platform for CHIKV. FLISA is a high-throughput detection method based on fluorescence signals, utilizing specific monoclonal antibodies-such as 2B5 and 2C8 targeting the CHIKV E1 protein-conjugated with coumarin-derived fluorophores. Compared with conventional ELISA, FLISA offers a lower analytical limit of detection, performing particularly well in human serum and blood samples. The analytical limit of detection of FLISA is 1 × 105 PFU/mL, which is twofold lower than that of ELISA, and it exhibits reduced interference from serum and blood components (Nguyen et al., 2019).

## 4 Advances in molecular diagnostic technologies for chikungunya virus

### 4.1 Gold standard and limitations

Through real-time amplification, RT-qPCR provides sensitive and specific detection of CHIKV, making it a cornerstone technique in molecular diagnostics. RT-qPCR detects CHIKV through reverse transcription of viral RNA into complementary DNA (cDNA), followed by real-time amplification and quantification using fluorescent probes. Depending on the workflow, RT-qPCR may be performed in either a one -step or two-step format. Since the resulting fluorescence increases in direct proportion to the amount of target DNA, the technique provides a reliable measure of viral load. Its high analytical sensitivity and specificity enable the detection of even low-level viral RNA (Andrew et al., 2023). For CHIKV detection, optimized RT-qPCR assays targeting the nsP1 or E1 genes have been shown to achieve analytical limits of detection as low as < 10 RNA copies/μL, and in some cases, single-copy detection has been reported. As a standard clinical method, RT-qPCR provides highly specific and sensitive detection of CHIKV (Wesselmann et al., 2024, 2025). Nevertheless, this approach still faces certain limitations. When detecting low-concentration virus in large-scale mosquito samples, it requires optimization of both RNA extraction and amplification protocols (Tang et al., 2020). Critically, early RT-qPCR false-negative results are more likely when specimens are collected outside the principal viremic window or during periods of rapidly declining viral load; under these conditions, viral RNA detection may be limited (Sajith et al., 2025). Future development of portable RT-qPCR platforms could significantly enhance diagnostic efficiency in point-of-care and field settings. However, several technical challenges remain: the need for stable cold chains for reagent transport, the requirement for trained personnel even with simplified protocols, and the potential for false-negative results due to viral RNA degradation during sample transport. Recent advances in lyophilized reagents and sample-stabilizing buffers show promise in addressing these limitations. Multiplex RT-qPCR can detect several arboviruses at once-including dengue, chikungunya, and Zika viruses-which greatly increases testing efficiency. A key challenge with this approach, however, lies in designing primers and probes that avoid cross-reactivity during the multiplexed real-time amplification step (Belem et al., 2024; Hauner et al., 2025).

### 4.2 Isothermal amplification (LAMP/RPA)

Recent advances in nucleic acid detection have largely centered on enhanced forms of isothermal amplification. Both loop-mediated isothermal amplification (LAMP) and recombinase polymerase amplification (RPA) enable nucleic acid amplification under relatively simple conditions and can be coupled with rapid, field-friendly readouts without the need for expensive thermocyclers. The amplification products of LAMP can be directly visualized through fluorescence, turbidity, or colorimetric readouts, without the need for expensive instrumentation for signal detection (Novi et al., 2025).

Compared with PCR-based methods, isothermal amplification techniques are therefore more suitable for nucleic acid testing in resource-limited settings, particularly during outbreaks of infectious diseases. LAMP requires only a simple constant-temperature heating module (60–65 °C), without the need for expensive PCR instruments (Xu et al., 2024; Luan et al., 2025). LAMP amplification is completed in approximately 45 min, with total turnaround time from sample to result ranging from 40 min to 2 h—far faster than conventional laboratory methods (Kumar et al., 2022). The technology also demonstrates high analytical sensitivity, with reported limits of detection as low as single-digit to tens of viral RNA copies per microliter (Sridhar et al., 2024). The availability of lyophilized LAMP kits circumvents cold-chain logistics, while technical refinements coupled with chromogenic indicators permit visual readout of results. The LAMP reaction system generally shows better tolerance to certain sample-derived inhibitors than conventional PCR (Mori et al., 2021). Despite the potential for high inhibitor concentrations to compromise amplification efficiency, straightforward sample pretreatment strategies—including heating, dilution, or application of dedicated lysis buffers—are generally sufficient to mitigate this limitation, thereby making LAMP a viable option for CHIKV diagnosis in resource-limited settings (Pollak et al., 2023a,b).

RPA is another widely used isothermal amplification approach that operates at relatively low temperatures, typically around 37–42°C, with some formats capable of amplification at temperatures as low as 25 °C. This temperature profile allows RPA to be operated with minimal heating infrastructure and supports point-of-care or decentralized testing workflows. RPA products can be detected using endpoint readouts such as lateral-flow strips or colorimetric readouts, as well as fluorescence-based systems, enabling implementation with relatively limited instrumentation. In addition, RPA has also been reported to tolerate several common amplification inhibitors and in some applications can be combined with simple sample pretreatment to enable testing of crude or minimally processed specimens (Daher et al., 2016; Lobato and O'Sullivan, 2018). For CHIKV specifically, RT-RPA has demonstrated rapid detection with high analytical and clinical performance, supporting its potential as an alternative molecular approach for point-of-need testing (Patel et al., 2016).

### 4.3 Next gen sequencing (NGS)

In recent years, high-throughput sequencing (HTS) has become an essential tool for the discovery and investigation of arboviruses. This technology enables the parallel sequencing of millions to billions of DNA or RNA fragments, facilitating the rapid acquisition of extensive genomic information (Ramírez et al., 2020). With its high analytical sensitivity and broad applicability, HTS allows both the detection of known viruses and the identification of emerging viral species, providing a powerful approach for arbovirus surveillance (Fagre et al., 2019). Compared with conventional Sanger sequencing, HTS enables simultaneous detection of multiple viruses in host blood meals and the characterization of diverse viral populations within individual mosquito vectors (Tchouassi et al., 2024).

HTS also facilitates whole-genome sequencing and provides valuable insights into viral evolution, genetic diversity, and transmission dynamics. For dengue virus (DENV), optimized primer designs, serotype-specific primer panels, and shortened amplicons have enabled efficient recovery of complete viral genome sequences from clinical specimens (Su et al., 2022). Furthermore, whole-genome sequencing combined with phylogenetic analysis has been applied to investigate the origin, spread, and evolutionary dynamics of arboviruses, such as yellow fever virus (YFV) (Perez et al., 2025). In addition, metagenomic sequencing approaches allow unbiased characterization of viral genetic material from vector organisms, such as mosquitoes, without requiring prior knowledge of specific viral sequences, thereby expanding the ability to monitor arboviral diversity (Ramírez et al., 2020).

For CHIKV, sequencing-based approaches, including Sanger sequencing, next-generation sequencing, and nanopore sequencing, have been applied for viral genotype identification and mutation surveillance (Machado et al., 2019; de Oliveira et al., 2021; Sawal et al., 2021). These approaches have contributed to a better understanding of the relationship between CHIKV genetic variation and viral biological characteristics. For example, genomic analyses revealed that the E1-A226V mutation in the East-Central-South-African (ECSA) genotype enhances viral adaptation to *Aedes albopictus* mosquitoes, thereby increasing its transmission potential (Alagarasu et al., 2023; Xiao et al., 2025). More recently, next-generation sequencing has enabled real-time monitoring of CHIKV evolution and facilitated the identification of emerging viral variants during outbreaks (Umair et al., 2025).

Based on the discussions in Sections 4.1 to 4.3, it is clear that each technology possesses unique strengths and weaknesses. Table 1 provides a brief introduction to various diagnostic techniques. Figure 4 provides a systematic side-by-side comparison, highlighting that RT-qPCR serves as the clinical backbone, LAMP is ideal for rapid on-site detection, and NGS is indispensable for research monitoring and epidemic tracing. Notably, the potential advantage of NGS in analytical resolution should be confined to well-defined applications such as whole -genome analysis and detailed epidemiological tracking, rather than being generalized to routine clinical diagnosis. Moreover, these benefits come with practical limitations, including high instrument cost, complex data analysis, and longer experimental turnaround times.

**Table 1:** Comparison of diagnostic approaches by detection target, advantages, limitations, and approximate diagnostic windows.

Method	Detection target	Main advantages	Major limitations	Diagnostic window
Virus isolation	Infection virus	High specificity	Time-consuming, biosafety requirements	Acute
Antigen detection	Viral antigen	Rapid, simple	Sensitivity, limited detection window	Early acute
RT-qPCR	Viral RNA	Sensitive, specific	Sampling-window dependent	Acute
Serology	IgG/IgM	Useful in later phase	Cross-reactivity	Convalescent
NGS	Viral genome	Broad detection/ genomic information	Cost, analysis complexity	Acute/outbreak
LAMP/RPA	Viral nucleic acid	Rapid, low infrastructure	Assay optimization	Acute
CRISPR	Viral nucleic acid	Potentially high specificity, rapid turnaround	Clinical validation needed	Acute
Biosensors	Antigen/RNA/antibody	Portable, rapid	Matrix effects/ standardization	Potential POCT
POCT	Antigen/RNA/antibody	Accessible, rapid	Sensitivity/quality control	Field/community

Diagnostic windows are approximate and may vary with specimen type, sampling timing, viral load, and assay format; overlap between phases is common.

**Figure 4:**
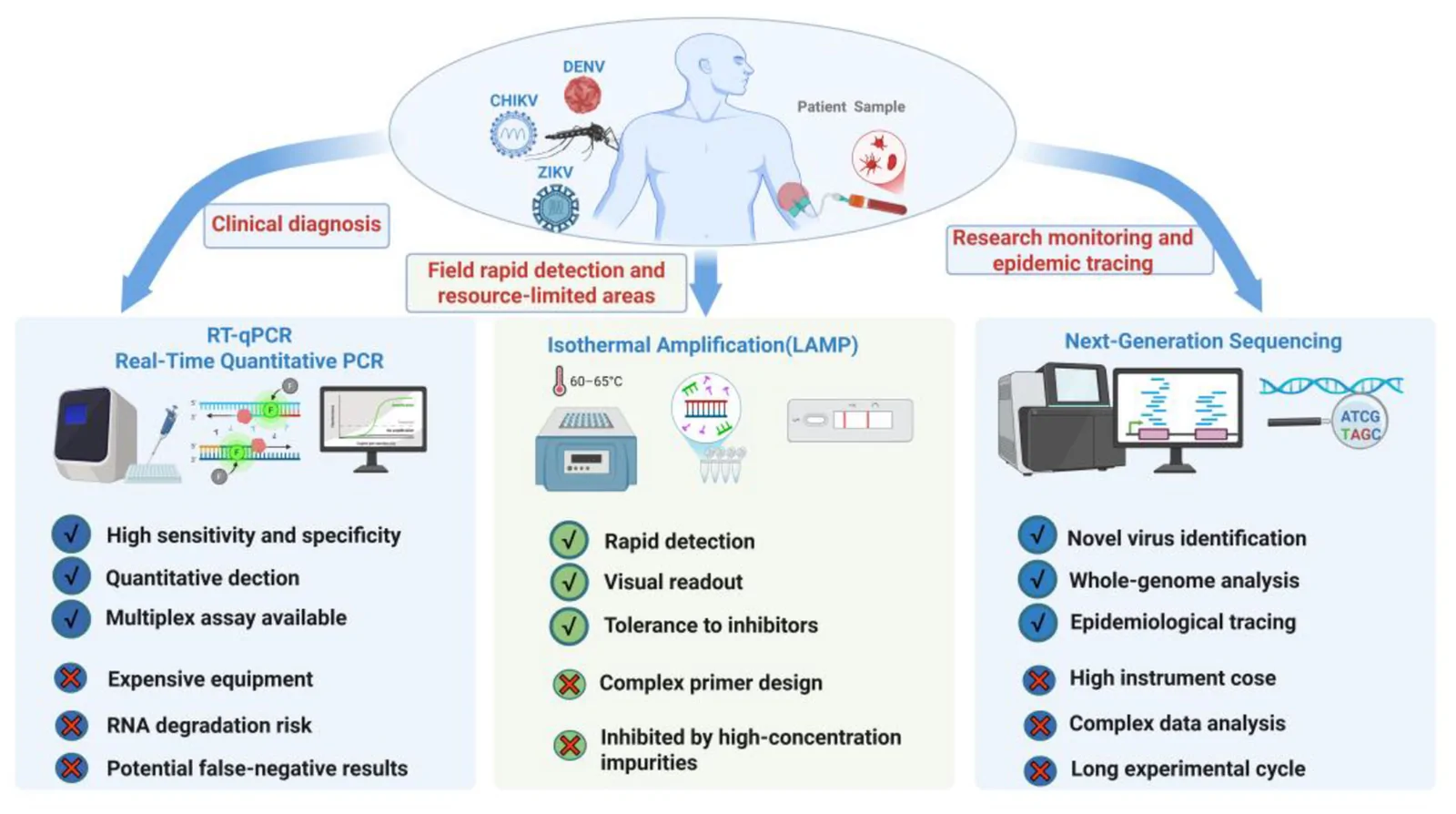
Comparison of representative methods: RT-qPCR, LAMP, and NGS for mosquito-borne arbovirus detection (CHIKV, DENV, and ZIKV). Schematic comparison of RT-qPCR, LAMP, and next-generation sequencing (NGS) for detection of CHIKV, DENV, and ZIKV. The figure summarizes typical strengths and limitations of each approach.

## 5 Arbovirus cross-reactivity challenges and E2-specific aptamer applications

### 5.1 DENV/ZIKV antibodies interfere with CHIKV detection

In regions where multiple arboviruses co-circulate, antibodies produced in response to previous DENV and ZIKV infections can significantly interfere with CHIKV serological detection, thereby reducing the specificity of serological assays. This interference is primarily mediated through various immunological mechanisms. Detailed information can be found in Table 2. Firstly, acute or recent flavivirus infections can induce a strong polyclonal antibody response, resulting in the production of large quantities of low-affinity IgM and IgG antibodies (Malafa et al., 2020), these antibodies may show imperfect antigen discrimination and cause non-specific binding in CHIKV ELISA or rapid diagnostic assays, which may lead to false-positive signals (Endale et al., 2021). Secondly, factors such as the host's prior infection history and circulating immune complexes can further affect the antigen-antibody interaction process, leading to false-positive signals or reduced assay specificity (Pezzi et al., 2019; Sena et al., 2024). Importantly, these effects may reflect non-specific binding, polyclonal heterologous immunity and assay-related limitations rather than true antigenic cross-reactivity at the epitope level. Although antibody-dependent enhancement (ADE) has been well described for some flaviviruses, its role in serological assay performance remains indirect and is largely speculative; therefore, it should not be used to directly explain diagnostic cross-reactivity (Bournazos et al., 2020). In summary, Diagnostic interference in co-circulating and prior-exposure settings can be mitigated by confirming CHIKV infection using complementary approaches such as nucleic acid testing or virus neutralization assays to improve overall diagnostic accuracy (Cardona-Trujillo et al., 2022; Kizu et al., 2024; Lin et al., 2024).

**Table 2:** Structural and functional comparison of CHIKV and other arboviruses.

Features	DENV	ZIKV	CHIKV
Family and genus	*Flaviviridae, Flavivirus*	*Flaviviridae, Flavivirus*	*Togaviridae, Alphavirus*
Morphology	The virion is roughly spherical, with a diameter of approximately 50 nm.	The virion is roughly spherical, with a diameter of approximately 50 nm.	The virion is roughly spherical, with a diameter of approximately 60–70 nm.
Genome type	Single-stranded positive-sense RNA (~10.7 kb)	Single-stranded positive-sense RNA (~10.8 kb)	Single-stranded positive-sense RNA (~11.8 kb)
Genome structure	A single open reading frame encoding three structural proteins (C, prM/M, and E) and seven non-structural proteins (NS1–NS5).	Similar to DENV, with minor differences in non-structural proteins.	Two major open reading frames encoding four non-structural proteins (nsP1–nsP4) and five structural proteins (C, E1, E2, E3, 6K/TF).
Capsid	Composed of the C Protein, enclosing the viral RNA.	Similar to DENV.	Composed of the C Protein, enclosing the RNA genome.
Envelope	Present, derived from the host membrane.	Present and derived from the host cell membrane.	Present, derived from the host cell membrane.
Surface glycoprotein	The E protein exists as dimers and mediates receptor binding and membrane fusion, while the prM/M protein participates in viral maturation.	The E protein forms homodimers and is highly homologous to that of DENV, although differences are present in specific loop regions. The pr M/M proteins exhibit similar structural features.	The E1 and E2 proteins form heterodimers, with E1 mediating membrane fusion and E2 responsible for receptor binding.
Structural symmetry	Icosahedral symmetry (T = 3)	Icosahedral symmetry (T = 3)	Icosahedral symmetry (T = 4)
Maturation process	Assembly in the endoplasmic reticulum → glycosylation and prM cleavage in the Golgi apparatus → secretion of mature virions	Similar to DENV	Assembly occurs at the inner side of the plasma membrane, with the transmembrane regions of E1/E2 anchoring the proteins, followed by release through budding.
Primary receptor(s)/Attachment factor(s)	DC-SIGN, TIM/TAM family members, and glycosaminoglycans	AXL, DC-SIGN, and members of the TIM/TAM family	MXRA8, glycosaminoglycans (GAGs), and DC-SIGN
Engages with host immunity	NS proteins suppress the interferon response, with NS5 targeting STAT2 for degradation.	Although similar to DENV, ZIKV NS5 displays enhanced affinity for human STAT2.	Nsp2 suppresses host transcription and disrupts the interferon-mediated antiviral response.
Antigenic similarity	High within flaviviruses	Strong similarity with DENV	Distinct from flaviviruses; conserved within alphaviruses
Serological cross-reactivity	Can contribute to misleading CHIKV serological signals (false-positive risk), depending on assay format and immune background (assay-dependent).	May contribute to heterologous/pseudo-cross-reactivity in co-endemic regions; magnitude depends on assay and timing.	True antigenic overlap with flaviviruses is generally lower than within flaviviruses, but reduced specificity can still occur due to immune background and assay-related non-specific binding.
Interference in CHIKV diagnosis	Pre-existing anti-DENV immunity may contribute to non-specific binding in CHIKV immunoassays, potentially producing false-positive IgM/IgG signals (assay and interpretation-dependent).	Anti-ZIKV immunity may contribute to pseudo-cross-reactivity in co-endemic settings, influencing serological interpretation depending on assay antigen design and timing.	Diagnostic accuracy is influenced by heterologous immunity and assay-related limitations.
Mechanistic basis	Heterologous immune background and assay-related non-specific binding (rather than confirmed epitope-level antigenic overlap).	Shared flavivirus immunogenicity can contribute to heterologous serological signals (assay-dependent).	Non-specific binding + immune background (not true antigenic overlap)

Despite the distinct taxonomic classification of CHIKV from flaviviruses such as Dengue virus and Zika virus, serological diagnosis is frequently confounded by immune-mediated interference rather than true antigenic cross-reactivity. Pre-existing flavivirus antibodies may induce non-specific binding in immunoassays, while repeated arboviral exposure generates a complex heterologous immune background. Consequently, diagnostic inaccuracies in CHIKV detection are largely driven by host immune history and assay design limitations, highlighting the need for integrated diagnostic strategies.

### 5.2 Specific antigen detection of E2 protein and the potential of aptamers

The E2 protein is a structural protein on the surface of the CHIKV virus, which is immunogenic and can be recognized by the host's immune system during the early stages of infection. It is also the main target of neutralizing antibodies (Andrew et al., 2025). Currently, most detection methods are still antibody-based, such as ELISA and immunofluorescence (Guo et al., 2022), these methods have achieved rapid detection of the E2 protein to some extent. However, in practical applications, they may still be affected by the antigenic similarity between arboviruses of the same genus (such as DENV and ZIKV), leading to cross-reactivity, which limits the improvement of specificity (Anh et al., 2025). Against this backdrop, aptamer technology has garnered increasing attention. Aptamers are short DNA or RNA sequences that fold into specific three-dimensional structures, enabling high-precision recognition and binding to target antigens. The CHIKV E2-targeting DNA aptamers, Chik-2 and Chik-3, demonstrated 80% sensitivity and 100% specificity in a sandwich ELISA setup (Andrew et al., 2025). This method is cost-effective and highly stable. Additionally, aptamers offer advantages such as controllable chemical synthesis, good batch-to-batch stability, and ease of modification. They can be integrated with platforms like electrochemical sensors, nanomaterials, or microfluidic chips to create novel detection systems, showing promising potential in terms of sensitivity and suitability for field applications, as shown in Figure 1 (Antigen detection) (Sharma et al., 2024; Kang et al., 2026; Sajikumar et al., 2026). Overall, targeting the E2 protein and incorporating aptamer strategies hold promise for achieving more stable and efficient CHIKV antigen detection, while simultaneously reducing cross-interference.

### 5.3 Cross-reactivity and diagnostic strategies for CHIKV differential diagnosis: emphasis on DENV, ZIKV

DENV and ZIKV both belong to the Flaviviridae family, and certain epitopes on their envelope (E) proteins are highly similar (Salgado et al., 2023; Elliott and Mattapallil, 2024). This shared antigenicity stems from their structural and genetic relatedness within the same viral family. In contrast, CHIKV, as a member of the *Togaviridae* family, possesses distinct structural proteins and genomic organization. A systematic comparison of the key virological characteristics among these arboviruses is summarized in Table 2. The occurrence of cross-reactivity can lead to false-positive results in serological assays, thereby affecting the interpretation of viral infection in the host.

To address this issue, researchers have developed serological detection methods based on non-structural protein 1 (NS1). The NS1-based DENV IgG ELISA can specifically detect DENV infection. Previous studies have used recombinant NS1 containing the C-terminal fragment with the most relevant B-cell epitopes for validation. Experimental results indicated a significant reduction in cross-reactivity between DENV and other flaviviruses (Pereira et al., 2021). Some studies have employed virus-like particles containing conserved residues on the envelope protein for ELISA. Researchers reported that key residues in the fusion loop (FL) and BC loop (BCL) of the DENV envelope protein are recognized by human monoclonal antibodies as well as polyclonal sera exhibiting flavivirus cross-reactivity. In a study involving 32 human monoclonal antibodies with flavivirus cross-reactivity, individual critical FL residues (W101 and F108) and BCL residues (T76, Q77, G78, E79) were identified as epitopes capable of effectively distinguishing serological cross-reactivity (Tsai et al., 2020). Researchers are continually developing monoclonal antibodies targeting unique epitopes of DENV NS1 for use in ELISA, immunochromatography, or protein microarray assays. These antibodies can minimize cross-reactivity with other flaviviruses, enabling pathogen-specific differential diagnosis (Thao et al., 2021). Furthermore, the Plaque Reduction Neutralization Test (PRNT) can be employed to mitigate the impact of viral cross-reactivity on diagnostic accuracy. As a highly specific confirmatory serological method, PRNT serves to detect and quantify virus-neutralizing antibodies. The underlying principle involves the incubation of the virus with serum samples prior to inoculation onto susceptible cell monolayers. The presence of neutralizing antibodies in the serum binds to the virus, preventing cellular infection and subsequently reducing or eliminating the formation of viral plaques within the cell culture (Ribeiro et al., 2020; Byaruhanga et al., 2023; Dia et al., 2023). In regions where multiple arboviruses co-circulate, PRNT is considered a robust method for differentiating viral infection status, offering high specificity and sensitivity (Blanco et al., 2024). By applying a 90% neutralization threshold, a more stringent criterion can be used to assess virus-specific neutralization and aid discrimination among related viruses (Ribeiro et al., 2020). In addition, the timing of sample collection, paired serum samples, molecular test results, exposure history, and comparative analyses involving related viruses remain important (Babu et al., 2023; Powers et al., 2023; Kizu et al., 2024). A multi-virus PRNT panel is used for simultaneous detection of multiple arboviruses, followed by comparison of neutralizing antibody titers to determine the viral infection status (Nunes et al., 2021; Orbegozo et al., 2025). Detecting neutralizing antibodies against these viruses is crucial for arbovirus epidemiological surveillance and effectively reduces the impact of cross-reactivity on diagnostic accuracy (Gyawali et al., 2020; Byaruhanga et al., 2023; de Lima et al., 2024; Dias et al., 2024). In the detection of CHIKV, studies have shown that the amino acid sequence of its E2B domain is highly conserved, and its unique antigenic epitopes can induce the production of a high proportion of neutralizing antibodies. Serological tests during the recovery phase indicate that this domain does not affect the neutralization of CHIKV itself but can reduce the titer of neutralizing antibodies against other viruses. This suggests that the E2B domain is a key target for cross-neutralization and may have protective cross-neutralizing antibodies (Powers et al., 2023). Furthermore, the CHIKV E2 domain mediates binding to the MXRA8 receptor, which facilitates viral entry into host cells (Zimmerman et al., 2023). However, receptor usage and viral entry tropism do not, by themselves, provide evidence for reduced serological cross-reactivity in diagnostic testing. In addition, serological cross-reactivity can also occur among related alphaviruses. Prior exposure to other arthritogenic alphaviruses may generate heterotypic antibody repertoires that recognize CHIKV antigens to varying degrees, depending on the antigen design and whether the assay is binding-based vs. neutralization-based (Powers et al., 2023; Kizu et al., 2024). Therefore, CHIKV serology in practice should be interpreted with consideration of potential heterologous alphavirus immunity, ideally supported by neutralization-based or orthogonal confirmatory testing (Weber et al., 2024).

## 6 Point-of-care testing

Although E2-specific aptamer-based strategies can improve the specificity of arbovirus detection, their application in clinical and field settings still faces several practical limitations. Most conventional diagnostic methods depend on well-equipped laboratories, specialized instruments, and trained personnel, which may limit their accessibility and delay diagnosis during arbovirus outbreaks, especially in regions with limited healthcare resources. Therefore, in addition to improving detection specificity, developing diagnostic approaches that are rapid, portable, affordable, and suitable for on-site testing has become increasingly important.

Point-of-care testing (POCT) provides a potential approach to address these challenges by allowing diagnostic procedures to be performed near patients or directly at outbreak sites without relying on centralized laboratory facilities. By combining simplified sample preparation, compact detection devices, and rapid signal analysis, POCT platforms can shorten turnaround times and facilitate timely decision-making. These characteristics make POCT particularly suitable for improving arbovirus surveillance and response, especially in areas where rapid and accessible diagnostic tools are urgently needed (Kumar et al., 2022; Plebani et al., 2025).

### 6.1 CRISPR-Cas-based molecular diagnostic technologies

With their notable specificity and sensitivity, CRISPR-Cas systems are increasingly being integrated into nucleic acid testing workflows. The integration of CRISPR-Cas12a with surface-enhanced Raman scattering (SERS) yields an amplification-free detection platform, achieving a reported analytical limit of detection lower (Yin et al., 2022). Integrating CRISPR-Cas13a with electrochemiluminescence (ECL) delivers RNA extraction and detection within 30 min and supports multiplex detection of several pathogens at once (Mao et al., 2025). Furthermore, the integration of CRISPR-Cas systems with microfluidic technology enables the miniaturization, automation, and integration of nucleic acid testing. The choice between Cas12 and Cas13 systems depends on the target nucleic acid: Cas12 recognizes DNA targets and exhibits trans-cleavage activity on single-stranded DNA reporters, while Cas13 targets RNA and cleaves RNA reporters. This supports in principle RNA-virus detection without reverse transcription; however, sensitive assays often require preamplification or signal enhancement, so claims of “direct” detection should be qualified. For arbovirus diagnostics, Cas13-based systems offer the advantage of direct RNA detection, potentially reducing assay complexity and turnaround time (Gootenberg et al., 2017; Li et al., 2024; Yang and Patel, 2024). However, there are also some challenges in POCT applications: although the CRISPR-Cas system can offer high analytical sensitivity, efficient nucleic acid extraction methods are still required to ensure sufficient target DNA recovery, especially for early-stage infections or samples with low viral loads. Additionally, clinical samples may contain PCR inhibitors, which can affect the efficiency of subsequent RPA and CRISPR reactions (Bhardwaj et al., 2024; Athipanyasilp et al., 2026). It is evident that nucleic acid extraction and purification face challenges in point-of-care testing. Furthermore, the detection results may be influenced by environmental and operational factors. Point-of-care testing technologies often lack real-time monitoring of internal reference genes (such as human RNase P) or positive controls, making it difficult to differentiate between true negatives and false negatives (Ma et al., 2023). Additionally, the multi-step operations of CRISPR in point-of-care testing require skilled personnel. In resource-limited areas, operational errors may prevent the expected results from being achieved (Bhardwaj et al., 2024; Zhuang et al., 2026).

### 6.2 Point-of-care (POC) rapid detection technologies

Microfluidic paper-based analytical devices (μ PADs) represent miniaturized paper-based platforms that exploit capillary forces to drive fluid movement. By integrating microfluidic techniques, μ PADs can perform sample separation, chemical reactions, and detection. The devices generally consist of multiple layers of paper, with specific regions allocated for sample loading, reagent reactions, and signal readout (Uhlikova et al., 2024). As a novel POCT approach, it is commonly integrated with immunological techniques, including enzyme-linked immunosorbent assays (ELISAs), or with molecular biology-based methods (Eivazzadeh-Keihan et al., 2019). A wax-printed paper-based platform has been developed to detect the non-structural protein NS1 of DENV and ZIKV. This device can detect viral protein concentrations down to 10 ng/mL in 6–8 min and facilitates result interpretation using a smartphone application (Bedin et al., 2017). In the detection of CHIKV, a paper-based device using laser-cut designs has been employed to measure CHIKV-specific IgM antibodies. The device leverages pseudovirus particles as capture antigens and completes testing in 10 min, with a reported sensitivity of 70.6% and specificity of 98%. Integrating biosensing with paper-based platforms allows for rapid, cost-effective viral detection and minimizes reliance on laboratory infrastructure, making it well-suited for field or remote applications (Theillet et al., 2019).

Rapid diagnostic tests (RDTs), as a form of POCT, enable the concurrent detection of multiple arboviruses, providing a clear advantage in the context of complex viral landscapes (Lee et al., 2021). Biosensor-based RDTs for DENV enable the concurrent detection of NS1 antigen and virus-specific antibodies (IgG/IgM), making them particularly useful for identifying acute dengue infections (Matusali et al., 2020). Nevertheless, the performance of RDTs is affected by the specific virus and the testing environment. Their application shows lower sensitivity when detecting CHIKV and ZIKV. For resource-limited settings where arboviruses are endemic, the ideal diagnostic platform should meet the ASSURED criteria (Affordable, Sensitive, Specific, User-friendly, Rapid and robust, Equipment-free, and Deliverable). Current RDTs partially meet these criteria but may sacrifice analytical sensitivity for simplicity. A tiered diagnostic approach may be optimal: initial screening with RDTs at peripheral health facilities, followed by confirmatory molecular testing at regional reference laboratories. This strategy balances the need for rapid results with diagnostic accuracy, while optimizing limited healthcare resources (Goh et al., 2022).

### 6.3 Multiplex detection

Since DENV, ZIKV, and CHIKV share the same mosquito vectors and are transmitted via mosquitoes—causing similar early clinical symptoms such as fever, rash, and joint pain—traditional single-target detection methods are prone to misdiagnosis (Raulino et al., 2021). Moreover, these three viruses often co-circulate in the same regions. Multiplex detection technologies can simultaneously detect DENV, ZIKV, and CHIKV in a single assay, significantly reducing the risk of misdiagnosis. This enables more efficient allocation of medical resources and provides a reliable tool for surveillance and control of these diseases (Gomgnimbou et al., 2024; Bandre et al., 2026). At the molecular diagnostic level, multiplex RT-qPCR can be employed, as shown in Figure 4. Different viruses are targeted using specific primers and probes labeled with distinct fluorescent reporter groups, enabling the simultaneous detection of multiple arboviruses in a single assay (Lura et al., 2022; Panmei et al., 2025). In addition, high-throughput sequencing enables comprehensive analysis of genetic information, effectively detecting viral profiles of CHIKV, DENV, and ZIKV. Its broader genomic information can complement RT-qPCR, making it a valuable tool for early warning and surveillance (de Araujo et al., 2025). In terms of serology, it is necessary to further develop detection methods targeting specific antigenic epitopes and to adopt combinatorial strategies to improve diagnostic accuracy.

## 7 Bioinformatics and artificial intelligence

Although conventional laboratory methods for viral detection are often time-consuming and require specialized facilities, recent advances in artificial intelligence (AI) have provided new opportunities for improving arbovirus diagnostics and surveillance. Machine learning and big data analytics have been increasingly integrated with biosensing platforms and epidemiological models to enhance detection accuracy and outbreak prediction. For example, machine learning-assisted optical and electrochemical immunosensors have demonstrated high sensitivity for DENV detection, with quantum dot- and antibody-based platforms showing excellent linearity across a broad concentration range (Torres do Couto et al., 2024). The combination of machine learning with near-infrared spectroscopy has also enabled rapid and accurate DENV identification (Goh et al., 2022). Beyond pathogen detection, AI-based models integrating mosquito surveillance, climatic variables, and human mobility data have shown strong potential for predicting arbovirus transmission risks, as demonstrated in West Nile virus (WNV) and dengue studies (Ramadona et al., 2023; Centrone et al., 2025; McMillan et al., 2025). Although AI applications in CHIKV diagnosis and surveillance remain relatively limited, these approaches provide valuable frameworks for future CHIKV monitoring by integrating molecular detection, ecological data, and epidemiological information.

## 8 Conclusion and future perspectives

CHIKV-relevant diagnostics have progressed substantially through advances in molecular biology, immunology, and bioengineering technologies. Conventional diagnostic approaches, including virus isolation, antigen detection, nucleic acid amplification, and serological assays, continue to serve as the foundation of CHIKV-relevant surveillance and clinical management. Taking CHIKV as a representative example, diagnostic strategies require careful consideration of viral kinetics and disease progression. Molecular assays, particularly RT-PCR-based methods, remain indispensable during the acute phase by enabling direct detection of viral RNA, whereas serological approaches provide complementary information during the later stages of infection. Meanwhile, emerging technologies, including next-generation sequencing, CRISPR-based detection platforms, and biosensing strategies, are extending CHIKV-relevant diagnostics beyond conventional laboratory-based testing by enabling broader pathogen identification and more flexible detection formats.

However, several critical challenges remain before these emerging technologies can achieve widespread clinical and field deployment. The extensive genetic diversity of arboviruses and the antigenic similarity among co-circulating viruses continue to complicate diagnostic accuracy, particularly for serological assays. Improving diagnostic specificity will require better characterization of viral antigenic determinants and host immune recognition patterns. Strategies including precise epitope characterization, rational antigen engineering, and the development of highly specific monoclonal antibody- or aptamer-based recognition systems may offer promising solutions to overcome cross-reactivity limitations. Furthermore, although advanced molecular and biosensing platforms have demonstrated excellent analytical performance, their dependence on sophisticated equipment and trained personnel may limit their application in resource-limited regions. These differences in diagnostic capacity across geographical regions reflect variation in laboratory infrastructure, healthcare resources, and access to advanced diagnostic platforms. High-resource settings generally have greater access to molecular, multiplex, automated, and sequencing-based technologies, whereas resource-limited and outbreak-prone regions may place greater emphasis on rapid, low-cost, and decentralized approaches such as rapid diagnostic tests and isothermal amplification. For CHIKV, these regional differences highlight the importance of developing diagnostic strategies that balance analytical performance with affordability, portability, operational simplicity, and local healthcare capacity.

Future efforts should therefore focus not only on improving analytical sensitivity and specificity but also on establishing clinical performance and facilitating the translation of innovative technologies into accessible diagnostic tools. Large-scale clinical validation using diverse patient cohorts, evaluation under real-world outbreak conditions, optimization of cost and operational requirements, and the establishment of standardized quality-control frameworks will be essential for successful implementation. In particular, integrating molecular diagnostics with portable platforms, digital technologies, and data-driven approaches may enable more efficient surveillance and early response to emerging CHIKV-relevant threats. Continued integration of virological knowledge with advances in diagnostic engineering and clinical validation will be essential for developing practical arbovirus detection systems.

## References

[B1] Afzal M. Sharma A. Sah A. K. Chandrasekar M. (2025). Evaluating anti-CHIK IgM and qualitative RT-PCR for diagnosing chikungunya in hospitalized patients: a comparative analysis of diagnostic efficacy. J. Vector Borne Dis. 62, 439–445. doi: 10.4103/jvbd.jvbd_125_24 40192149

[B2] Alagarasu K. Patil J. Jadhav S. Chowdhury D. Bote M. Punekar M. . (2023). Understanding the resurgence of chikungunya virus during 2020-2021 in Pune, India, based on genomic analyses: a seven year study. J. Med. Virol. 95:e29253. doi: 10.1002/jmv.29253 38009608

[B3] Alejo-Cancho I. Navero-Castillejos J. Peiró-Mestres A. Albarracín R. Barrachina J. Navarro A. . (2020). Evaluation of a novel microfluidic immuno-magnetic agglutination assay method for detection of dengue virus NS1 antigen. PLoS Negl. Trop. Dis. 14:e0008082. doi: 10.1371/journal.pntd.0008082 32069280 PMC7048294

[B4] Allen S. E. Jardine C. M. Hooper-McGrevy K. Ambagala A. Bosco-Lauth A. M. Kunkel M. R. . (2020). Serologic evidence of arthropod-borne virus infections in Wild and Captive Ruminants in Ontario, Canada. Am. J. Trop. Med. Hyg. 103, 2100–2107. doi: 10.4269/ajtmh.20-0539 32815506 PMC7646771

[B5] Amaral M. P. Coirada F. C. de Souza Apostolico J. Tomita N. Fernandes E. R. Santos Souza H. F. . (2021). Prime-boost with Chikungunya virus E2 envelope protein combined with Poly (I:C) induces specific humoral and cellular immune responses. Curr. Res. Immunol. 2, 23–31. doi: 10.1016/j.crimmu.2021.03.001 35492391 PMC9040086

[B6] Andrew A. Citartan M. Wong K. A. Tang T. H. Magdline Sia Henry S. Ch'ng E. S. (2023). Analytical and clinical evaluation of a TaqMan Real-Time PCR assay for the detection of Chikungunya virus. Microbiol. Spectr. 11, e00088–23. doi: 10.1128/spectrum.00088-23 37272795 PMC10433969

[B7] Andrew A. Navien T. N. Yeoh T. S. Citartan M. Mangantig E. Sum M. S. H. . (2022). Diagnostic accuracy of serological tests for the diagnosis of Chikungunya virus infection: a systematic review and meta-analysis. PLoS Negl. Trop. Dis. 16:e0010152. doi: 10.1371/journal.pntd.0010152 35120141 PMC8849447

[B8] Andrew A. Sum M. S. H. Ch'ng E. S. Tang T.-H. Citartan M. (2025). Selection of DNA aptamers against Chikungunya virus Envelope 2 Protein and their application in sandwich ELASA. Talanta 281:126842. doi: 10.1016/j.talanta.2024.126842 39305759

[B9] Anh D. D. Loc D. H. Tong H. V. Dieu D. T. H. Hang N. T. Lanh N. H. . (2025). Dominance of DENV-1 and flavivirus serological cross-reactivity during the 2016 dengue outbreak in Vietnam. J. Med. Virol. 97:e70569. doi: 10.1002/jmv.70569 40838976 PMC12369675

[B10] Aranda-Coello J. M. Machain-Williams C. Weber M. Dzul Rosado A. R. Simpkins T. R. Blitvich B. J. (2025). Serologic surveillance for orthoflaviviruses and chikungunya virus in Bats and Opossums in Chiapas, Mexico. Viruses 17:590. doi: 10.3390/v17050590 40431603 PMC12115384

[B11] Athipanyasilp N. Saowpak S. Chaimayo C. Angkasekwinai N. Pattama A. Athipanyasilp A. . (2026). CRISPR-Cas13a SHERLOCK assay for rapid and sensitive detection of chikungunya virus. Microbiol. Spectr. 14:e0229825. doi: 10.1128/spectrum.02298-25 41416796 PMC12889125

[B12] Aurelio B. Kaisar M. M. M. Wijaya V. Khustiawan S. O. R. Hengestu A. Ali S. (2026). A systematic review and meta-analysis of global DENV, CHIKV, and ZIKV Co-infection. Rev. Med. Virol. 36:e70108. doi: 10.1002/rmv.70108 41619199

[B13] Babu N. Mahilkar S. Jayaram A. Ibemgbo S. A. Mathur G. Shetty U. . (2023). Cytokine profile, neutralisation potential and viral replication dynamics in sera of chikungunya patients in India: a cross-sectional study. Lancet Reg. Health Southeast Asia 19:100269. doi: 10.1016/j.lansea.2023.100269 38076718 PMC10709682

[B14] Badar N. Ikram A. Salman M. Alam M. M. Umair M. Arshad Y. . (2021). Chikungunya virus: molecular epidemiology of nonstructural proteins in Pakistan. PLoS ONE 16:e0260424. doi: 10.1371/journal.pone.0260424 34941888 PMC8699639

[B15] Baer A. Kehn-Hall K. (2014). Viral concentration determination through plaque assays: using traditional and novel overlay systems. J. Vis. Exp. 93:e52065. doi: 10.3791/52065-v 25407402 PMC4255882

[B16] Bandre G. R. Bankar N. J. Dawande P. Ugemuge S. (2026). Advances in RT-PCR-based point-of-care systems for rapid detection of dengue virus serotypes: a narrative review. J. Vector Borne Dis. doi: 10.4103/jvbd.jvbd_164_25. [Epub ahead of print]. 41808354

[B17] Basore K. Kim A. S. Nelson C. A. Zhang R. Smith B. K. Uranga C. . (2019). Cryo-EM structure of chikungunya virus in complex with the Mxra8 receptor. Cell 177, 1725–1737.e16. doi: 10.1016/j.cell.2019.04.006 31080061 PMC7227486

[B18] Bedin F. Boulet L. Voilin E. Theillet G. Rubens A. Rozand C. (2017). Paper-based point-of-care testing for cost-effective diagnosis of acute flavivirus infections. J. Med. Virol. 89, 1520–1527. doi: 10.1002/jmv.24806 28295400

[B19] Belem L. R. W. Ibemgbo S. A. Gomgnimbou M. K. Verma D. K. Kaboré A. Kumar A. . (2024). Development of multiplex molecular assays for simultaneous detection of dengue serotypes and chikungunya virus for arbovirus surveillance. Curr. Issues Mol. Biol. 46, 2093–2104. doi: 10.3390/cimb46030134 38534750 PMC10969301

[B20] Bhardwaj P. Gulafshan S. Singh R. (2024). A rapid, specific and ultrasensitive detection of the Chikungunya virus based on RT-RPA:CRISPR/Cas12a one-pot dual mode end-point detection system. Anal. Chim. Acta 1329:343221. doi: 10.1016/j.aca.2024.343221 39396286

[B21] Blanco S. Marín Á. L. Frutos M. C. Barahona N. Y. Rivarola M. E. Carrizo L. H. . (2024). Haemovigilance survey and screening strategy for arthropod-borne viruses in blood donors from Argentina. J. Med. Virol. 96:e29476. doi: 10.1002/jmv.29476 38373210

[B22] Bournazos S. Gupta A. Ravetch J. V. (2020). The role of IgG Fc receptors in antibody-dependent enhancement. Nat. Rev. Immunol. 20, 633–643. doi: 10.1038/s41577-020-00410-0 32782358 PMC7418887

[B23] Broeders S. Garlant L. Fraiture M.-A. Vandermassen E. Suin V. Vanhomwegen J. . (2020). A new multiplex RT-qPCR method for the simultaneous detection and discrimination of Zika and chikungunya viruses. Int. J. Infect. Dis. 92, 160–170. doi: 10.1016/j.ijid.2019.12.028 31884173 PMC7129992

[B24] Byaruhanga T. Kayiwa J. T. Nankya A. M. Ataliba I. J. McClure C. P. Ball J. K. . (2023). Arbovirus circulation, epidemiology and spatiotemporal distribution in Uganda. IJID Reg. 6, 171–176. doi: 10.1016/j.ijregi.2023.01.013 36915800 PMC10006739

[B25] Cardona-Trujillo M. C. Ocampo-Cárdenas T. Tabares-Villa F. A. Zuluaga-Vélez A. Sepúlveda-Arias J. C. (2022). Recent molecular techniques for the diagnosis of Zika and Chikungunya infections: a systematic review. Heliyon 8:e10225. doi: 10.1016/j.heliyon.2022.e10225 36033321 PMC9404361

[B26] Carneiro P. H. Mendes-Monteiro L. Mohana-Borges R. (2022). Virus propagation and plaque assay for dengue virus. Methods Mol. Biol. 2409, 3–9. doi: 10.1007/978-1-0716-1879-0_1 34709631

[B27] Carrillo F. A. B. Ojeda S. Sanchez N. Plazaola M. Collado D. Miranda T. . (2025). Comparison of dengue, chikungunya, and Zika among children in Nicaragua across 18 years: a single-centre, prospective cohort study. Lancet Child Adolesc. Health 9, 622–633. doi: 10.1016/S2352-4642(25)00168-3 40774783 PMC12325154

[B28] Centrone F. Procacci R. Melilli R. Orlando V. A. Colella V. Loconsole D. . (2025). First seroprevalence study of West Nile Virus (WNV) infection in blood donors after the upsurge of West Nile Neuroinvasive Disease (WNND) cases in southern Italy in 2023. BMC Infect. Dis 25:200. doi: 10.1186/s12879-025-10603-4 39930346 PMC11812252

[B29] Cherabuddi K. Iovine N. M. Shah K. White S. K. Paisie T. Salemi M. . (2016). Zika and Chikungunya virus co-infection in a traveller returning from Colombia, 2016: virus isolation and genetic analysis. JMM Case Rep. 3:e005072. doi: 10.1099/jmmcr.0.005072 28348794 PMC5343122

[B30] Cho S.-Y. Lee D.-G. Park J. Y. Kim W.-B. Lee R. Nho D. . (2024). Predicting immunogenic epitopes variation of envelope 2 gene among chikungunya virus clonal lineages by an in silico approach. Viruses 16:1689. doi: 10.3390/v16111689 39599804 PMC11599094

[B31] Choudhary S. Neetu N. Singh V. A. Kumar P. Chaudhary M. Tomar S. (2021). Chikungunya virus titration, detection and diagnosis using N-Acetylglucosamine (GlcNAc) specific lectin based virus capture assay. Virus Res. 302:198493. doi: 10.1016/j.virusres.2021.198493 34175343

[B32] Daher R. K. Stewart G. Boissinot M. Bergeron M. G. (2016). Recombinase polymerase amplification for diagnostic applications. Clin. Chem. 62, 947–958. doi: 10.1373/clinchem.2015.245829 27160000 PMC7108464

[B33] Dai Y. Li Z. Zhang X. Gu Q. Xu J. Chu M. (2026). Chikungunya: global epidemiology, clinical spectrum, and vector control-lessons for China from the 2025 Guangdong outbreak. Arch. Toxicol. 100, 1225–1239. doi: 10.1007/s00204-025-04273-z 41535585

[B34] de Araujo J. C. Carvalho A. P. A. Adelino T. Iani F. C. M. Guimaraes N. R. Santos S. C. F. . (2025). Uncovering DENV, CHIKV, and ZIKV in Urban Wastewater in Brazil through genomic and molecular screening. Microorganisms 13:2164. doi: 10.3390/microorganisms13092164 41011495 PMC12472667

[B35] de Lima Cavalcanti T. Y. V. Pereira M. R. de Paula S. O. de Oliveira Franca R. F. (2022). A review on chikungunya virus epidemiology, pathogenesis and current vaccine development. Viruses 14:969. doi: 10.3390/v14050969 35632709 PMC9147731

[B36] de Lima R. C. Dias H. G. de Souza T. M. A. Familiar-Macedo D. Ribeiro E. D. Corrêa V. C. E. . (2024). Oropouche virus exposure in febrile patients during chikungunya virus introduction in the State of Amapá, Amazon Region, Brazil. Pathogens 13:469. doi: 10.3390/pathogens13060469 38921767 PMC11206884

[B37] de Oliveira E. C. Fonseca V. Xavier J. Adelino T. Morales Claro I. Fabri A. . (2021). Short report: introduction of chikungunya virus ECSA genotype into the Brazilian Midwest and its dispersion through the Americas. PLoS Negl. Trop. Dis. 15:e0009290. doi: 10.1371/journal.pntd.0009290 33861753 PMC8051810

[B38] de Souza W. M. Lecuit M. Weaver S. C. (2025). Chikungunya virus and other emerging arthritogenic alphaviruses. Nat. Rev. Microbiol. 23, 585–601. doi: 10.1038/s41579-025-01177-8 40335675

[B39] Dia M. Bob N. S. Talla C. Dupressoir A. Escadafal C. Thiam M. S. . (2023). Performance assessment and validation of a plaque reduction neutralization test (PRNT) in support to yellow fever diagnostic and vaccine clinical trials. J. Med. Virol. 95:e28700. doi: 10.1002/jmv.28700 36951314

[B40] Dias H. G. Familiar-Macedo D. Garrido I. O. Dos Santos F. B. Pauvolid-Corrêa A. (2024). Exposure of domestic animals to Mayaro and Oropouche viruses in urban and peri-urban areas of West-Central Brazil. One Health Outlook 6:12. doi: 10.1186/s42522-024-00104-w 38946009 PMC11215823

[B41] Eivazzadeh-Keihan R. Pashazadeh-Panahi P. Mahmoudi T. Chenab K. K. Baradaran B. Hashemzaei M. . (2019). Dengue virus: a review on advances in detection and trends - from conventional methods to novel biosensors. Mikrochim. Acta 186:329. doi: 10.1007/s00604-019-3420-y 31055654

[B42] Elliott K. C. Mattapallil J. J. (2024). Zika Virus-A reemerging neurotropic arbovirus associated with adverse pregnancy outcomes and neuropathogenesis. Pathogens 13:177. doi: 10.3390/pathogens13020177 38392915 PMC10892292

[B43] Endale A. Medhin G. Darfiro K. Kebede N. Legesse M. (2021). Magnitude of antibody cross-reactivity in medically important mosquito-borne Flaviviruses: a systematic review. Infect. Drug. Resist. 14, 4291–4299. doi: 10.2147/IDR.S336351 34703255 PMC8541746

[B44] Fagre A. C. Lee J. S. Kityo R. M. Bergren N. A. Mossel E. C. Nakayiki T. . (2019). Discovery and characterization of Bukakata orbivirus (Reoviridae:Orbivirus), a Novel Virus from a Ugandan Bat. Viruses 11:209. doi: 10.3390/v11030209 30832334 PMC6466370

[B45] Fan N. Sun D. W. Cheng R. Fu S. H. Zeng L. H. Wu Q. . (2020). [Isolation and identification of arbovirus in Hainan province, 2017–2018]. Zhonghua Liu Xing Bing Xue Za Zhi 41, 236–243. 10.3760/cma.j.issn.0254-6450.2020.02.018 32164136

[B46] Ferdous J. Nasif M. A. O. Cowman G. Muntasir I. Hassan M. R. Qayum M. O. . (2026). Zika virus outbreak — Bangladesh, September–December 2024. MMWR Morb. Mortal. Wkly. Rep. 75, 1–6. doi: 10.15585/mmwr.mm7501a1 41505349 PMC12782236

[B47] Freppel W. Silva L. A. Stapleford K. A. Herrero L. J. (2024). Pathogenicity and virulence of chikungunya virus. Virulence 15:2396484. doi: 10.1080/21505594.2024.2396484 39193780 PMC11370967

[B48] Fumagalli M. J. de Souza W. M. Espósito D. L. A. Silva A. Romeiro M. F. Martinez E. Z. . (2018). Enzyme-linked immunosorbent assay using recombinant envelope protein 2 antigen for diagnosis of Chikungunya virus. Virol J. 15:112. doi: 10.1186/s12985-018-1028-1 30041676 PMC6056935

[B49] Goh B. Visendi P. Lord A. R. Ciocchetta S. Liu W. Sikulu-Lord M. T. (2022). First report of the detection of DENV1 in human blood plasma with near-infrared spectroscopy. Viruses 14:2248. doi: 10.3390/v14102248 36298803 PMC9609583

[B50] Gomgnimbou M. K. Belem L. R. W. Bilgo E. Amara M. F. Laouali Z. Ouari A. . (2024). Potential performance of two new RT-PCR and RT-qPCR methods for multiplex detection of dengue virus Serotypes 1–4 and Chikungunya virus in mosquitoes. CIMB 46, 11048–11056. doi: 10.3390/cimb46100656 39451536 PMC11505945

[B51] Gootenberg J. S. Abudayyeh O. O. Lee J. W. Essletzbichler P. Dy A. J. Joung J. . (2017). Nucleic acid detection with CRISPR-Cas13a/C2c2. Science 356, 438–442. doi: 10.1126/science.aam9321 28408723 PMC5526198

[B52] Goto K. Amano R. Ichinose A. Michishita A. Hamada M. Nakamura Y. . (2025). Generation of RNA aptamers against chikungunya virus E2 envelope protein. J. Virol. 99:e0209524. doi: 10.1128/jvi.02095-24 39927773 PMC11915788

[B53] Guo M. Du S. Lai L. Wu W. Huang X. Li A. . (2022). Development and evaluation of recombinant E2 protein based IgM capture enzyme-linked immunosorbent assay (ELISA) and double antigen sandwich ELISA for detection of antibodies to Chikungunya virus. PLoS Negl. Trop. Dis. 16:e0010829. doi: 10.1371/journal.pntd.0010829 36480572 PMC9767333

[B54] Gyawali N. Murphy A. K. Hugo L. E. Devine G. J. (2020). A micro-PRNT for the detection of Ross River virus antibodies in mosquito blood meals: a useful tool for inferring transmission pathways. PLoS ONE 15:e0229314. doi: 10.1371/journal.pone.0229314 32706777 PMC7380888

[B55] Hamrahjoo M. Shams F. Saadat N. Marhamati S. Teimoori A. (2026). Overview of Chikungunya virus pathogenesis, genome variation, epidemiology, and control. Virus Res. 366:199703. doi: 10.1016/j.virusres.2026.199703 41722616 PMC12964048

[B56] Hatami J. de Bruin A. C. M. Bánki Z. Rey F. A. Gerold G. (2026). Antibody cross-reactivity among alphaviruses in clinical practice. Trends Microbiol. 34, 194–206. doi: 10.1016/j.tim.2025.07.011 40866148

[B57] Hauner A. Rogé S. Vanlerberghe V. Lepore L. Ndayisenga F. Shyaka A. . (2025). Technical validation of a multiplex real-time PCR for combined detection of Rift Valley fever, chikungunya, Zika and dengue viruses. J. Virol. Methods 337:115174. doi: 10.1016/j.jviromet.2025.115174 40345597 PMC12328903

[B58] Hernandez R. Brown D. T. Paredes A. (2014). Structural differences observed in arboviruses of the alphavirus and flavivirus genera. Adv. Virol. 2014, 1–24. doi: 10.1155/2014/259382 25309597 PMC4182009

[B59] Holmes A. C. Basore K. Fremont D. H. Diamond M. S. (2020). A molecular understanding of alphavirus entry. PLoS Pathog. 16:e1008876. doi: 10.1371/journal.ppat.1008876 33091085 PMC7580943

[B60] Hoornweg T. E. van Duijl-Richter M. K. S. Ayala Nuñez N. V. Albulescu I. C. van Hemert M. J. Smit J. M. (2016). Dynamics of Chikungunya virus cell entry unraveled by single-virus tracking in living cells. J. Virol. 90, 4745–4756. doi: 10.1128/JVI.03184-15 26912616 PMC4836339

[B61] Jiang M. Wan M. Fan Q. Min Y. Tang G. Wen Y. . (2024). Full genomic sequence characterization of the chikungunya virus from an imported case with serum viral concentration below culturable level. Biosaf. Health 6, 304–309. doi: 10.1016/j.bsheal.2024.09.003 40078739 PMC11894991

[B62] Jibowu M. Vigilant M. Costa-da-Silva A. L. Nelson A. Essigmann H. T. Brown E. L. . (2025). Spatial and seasonal dynamics of mosquito species in Harris County, Texas, highlight risk of arbovirus introduction and transmission. Sci. Rep. 15:10330. doi: 10.1038/s41598-025-92175-6 40133319 PMC11937572

[B63] Johnson B. W. Russell B. J. Goodman C. H. (2016). Laboratory diagnosis of chikungunya virus infections and commercial sources for diagnostic assays. J. Infect. Dis. 214, S471–S474. doi: 10.1093/infdis/jiw274 27920176 PMC5657184

[B64] Kang H. Auzenbergs M. Clapham H. Maure C. Kim J.-H. Salje H. . (2024). Chikungunya seroprevalence, force of infection, and prevalence of chronic disability after infection in endemic and epidemic settings: a systematic review, meta-analysis, and modelling study. Lancet Infect. Dis. 24, 488–503. doi: 10.1016/S1473-3099(23)00810-1 38342105

[B65] Kang J. Park J. Kim D. H. Park S. Baek S. Kim C. . (2026). A portable surface-free electrochemical aptamer-based biosensor for multiplex detection of arboviruses. Anal. Chem. 98, 9851–9860. doi: 10.1021/acs.analchem.5c07608 41882995

[B66] Kasbergen L. M. R. De Bruin E. Chandler F. Sigfrid L. Chan X. H. S. Hookham L. . (2025). Multi-antigen serology and a diagnostic algorithm for the detection of arbovirus infections as novel tools for arbovirus preparedness in southeast Europe (MERMAIDS-ARBO): a prospective observational study. Lancet Infect. Dis. 25, 678–689. doi: 10.1016/S1473-3099(24)00654-6 39987930

[B67] Kasbergen L. M. R. Nieuwenhuijse D. F. de Bruin E. Sikkema R. S. Koopmans M. P. G. (2023). The increasing complexity of arbovirus serology: an in-depth systematic review on cross-reactivity. PLoS Negl. Trop. Dis. 17:e0011651. doi: 10.1371/journal.pntd.0011651 37738270 PMC10550177

[B68] Khongwichit S. Chuchaona W. Korkong S. Wongsrisang L. Thongmee T. Poovorawan Y. (2025). Chikungunya virus in Thailand (2020–2023): epidemiology, clinical features, and genomic insights. PLoS Negl. Trop. Dis. 19:e0013548. doi: 10.1371/journal.pntd.0013548 40953104 PMC12449006

[B69] Kizu J. Graham M. Liu W. (2024). Potential serological misdiagnosis of Barmah Forest virus and Ross River virus diseases as Chikungunya virus infections in Australia: comparison of ELISA with neutralization assay results. Viruses 16:384. doi: 10.3390/v16030384 38543750 PMC10974935

[B70] Kumar A. Parihar A. Panda U. Parihar D. S. (2022). Microfluidics-Based Point-of-Care Testing (POCT) devices in dealing with waves of COVID-19 pandemic: the emerging solution. ACS Appl. Bio. Mater. 5, 2046–2068. doi: 10.1021/acsabm.1c01320 35473316

[B71] Kumar S. Sharma S. Kumari S. Pande V. Savargaonkar D. Anvikar A. R. (2022). Magnetic Multiplex Loop Mediated Isothermal Amplification (MM-LAMP) technique for simultaneous detection of dengue and chikungunya virus. J. Virol. Methods 300:114407. doi: 10.1016/j.jviromet.2021.114407 34896457

[B72] Lee W. Kim H. Bae P. K. Lee S. Yang S. Kim J. (2021). A single snapshot multiplex immunoassay platform utilizing dense test lines based on engineered beads. Biosens. Bioelectron. 190:113388. doi: 10.1016/j.bios.2021.113388 34098362 PMC8166042

[B73] Li S.-Y. Cheng Q.-X. Liu J.-K. Nie X.-Q. Zhao G.-P. Wang J. (2024). Author correction: CRISPR-Cas12a has both cis- and trans-cleavage activities on single-stranded DNA. Cell. Res. 34, 266–267. doi: 10.1038/s41422-024-00927-2 38263279 PMC10907588

[B74] Li X. Wan X. Liu J. Wang H. Li A. Ke C. . (2022). Luciferase immunosorbent assay based on multiple E antigens for the detection of Chikungunya virus-specific IgG antibodies. Microbiol. Spectr. 10:e0149621. doi: 10.1128/spectrum.02282-22 35311573 PMC9045172

[B75] Lin H.-C. Chang S.-F. Su C.-L. Hu H.-C. Chiao D.-J. Hsu Y.-L. . (2024). Facile quantitative diagnostic testing for neutralizing antibodies against Chikungunya virus. BMC Infect. Dis. 24:1076. doi: 10.1186/s12879-024-09973-y 39350079 PMC11440707

[B76] Liu J. Quan Y. Tong H. Zhu Y. Shi X. Liu Y. . (2024). Insights into mosquito-borne arbovirus receptors. Cell Insight 3:100196. doi: 10.1016/j.cellin.2024.100196 39391003 PMC11462183

[B77] Lobato I. M. O'Sullivan C. K. (2018). Recombinase polymerase amplification: basics, applications and recent advances. Trends Analyt. Chem. 98, 19–35. doi: 10.1016/j.trac.2017.10.015 32287544 PMC7112910

[B78] Louis R. Moquin T. L. Mavian C. Barthelemy A. Pu R. Anderson B. . (2025). Dengue and other arbovirus infections among Schoolchildren, Haiti, 2021. Emerg. Infect. Dis. 31, 336–340. doi: 10.3201/eid3102.240791 39983707 PMC11845146

[B79] Luan J. Song S. Cheng C. Li D. Zhu L. Cheng H. . (2025). Development of a capillary-modified naked-eye visual loop-mediated isothermal amplification method for the rapid detection of mpox virus and chikungunya virus. Front. Microbiol. 16:1615132. doi: 10.3389/fmicb.2025.1615132 40838013 PMC12362987

[B80] Lura T. Su T. Thieme J. Brown M. Q. (2022). A validated triplex RT-qPCR protocol to simultaneously detect chikungunya, dengue and Zika viruses in mosquitoes. J. Vector Borne Dis. 59, 198–205. doi: 10.4103/0972-9062.316275 36511035

[B81] Ma Y. Wu H. Chen S. Xie C. Hu J. Qi X. . (2023). FEN1-aided recombinase polymerase amplification (FARPA) for one-pot and multiplex detection of nucleic acids with an ultra-high specificity and sensitivity. Biosens. Bioelectron. 237:115456. doi: 10.1016/j.bios.2023.115456 37354713

[B82] Machado L. C. De Morais-Sobral M. C. Campos T. D. L. Pereira M. R. De Albuquerque M. D. F. P. M. Gilbert C. . (2019). Genome sequencing reveals coinfection by multiple chikungunya virus genotypes in a recent outbreak in Brazil. PLoS Negl. Trop. Dis. 13:e0007332. doi: 10.1371/journal.pntd.0007332 31095561 PMC6541278

[B83] Malafa S. Medits I. Aberle J. H. Aberle S. W. Haslwanter D. Tsouchnikas G. . (2020). Impact of flavivirus vaccine-induced immunity on primary Zika virus antibody response in humans. PLoS Negl. Trop. Dis. 14:e0008034. doi: 10.1371/journal.pntd.0008034 32017766 PMC7021315

[B84] Mangala Prasad V. Blijleven J. S. Smit J. M. Lee K. K. (2022). Visualization of conformational changes and membrane remodeling leading to genome delivery by viral class-II fusion machinery. Nat. Commun. 13:4772. doi: 10.1038/s41467-022-32431-9 35970990 PMC9378758

[B85] Mao X. Lu Y. Gao Z. Zhong J. Xiao A. Lin J. . (2025). Modular microfluidic sensor integrating nucleic acid extraction, CRISPR/Cas13a, and electrochemiluminescence for multichannel RNA detection. Anal. Chem. 97, 5085–5092. doi: 10.1021/acs.analchem.4c06197 40019770

[B86] Marfía J. I. Smith I. Targovnik A. M. Di Lello F. A. Wolman F. J. Flichman D. M. . (2025). Development and validation of dengue virus envelope protein domain III IgG antibody enzyme-linked immunosorbent assay. J. Immunol. Methods 542:113887. doi: 10.1016/j.jim.2025.113887 40447073

[B87] Martins E. B. Silva M. F. B. Tassinari W. S. de Bruycker-Nogueira F. Moraes I. C. V. Rodrigues C. D. S. . (2022). Detection of Chikungunya virus in bodily fluids: the INOVACHIK cohort study. PLoS Negl. Trop. Dis. 16:e0010242. doi: 10.1371/journal.pntd.0010242 35255099 PMC8929700

[B88] Matusali G. Colavita F. Carletti F. Lalle E. Bordi L. Vairo F. . (2020). Performance of rapid tests in the management of dengue fever imported cases in Lazio, Italy 2014-2019. Int. J. Infect. Dis. 99, 193–198. doi: 10.1016/j.ijid.2020.07.008 32659449

[B89] McMillan J. R. Sun J. Chaves L. F. Armstrong P. M. (2025). Using mosquito and arbovirus data to computationally predict West Nile virus in unsampled areas of the Northeast United States. PNAS Nexus 4:pgaf227. doi: 10.1093/pnasnexus/pgaf227 40838020 PMC12362355

[B90] Mendoza E. J. Manguiat K. Wood H. Drebot M. (2020). Two detailed plaque assay protocols for the quantification of infectious SARS-CoV-2. Curr. Protoc. Microbiol. 57:cpmc105. doi: 10.1002/cpmc.105 32475066 PMC7300432

[B91] Miller J. R. Koren S. Dilley K. A. Puri V. Brown D. M. Harkins D. M. . (2018). Analysis of the Aedes albopictus C6/36 genome provides insight into cell line utility for viral propagation. Gigascience 7, 1–13. doi: 10.1093/gigascience/gix135 29329394 PMC5869287

[B92] Mori A. Pomari E. Deiana M. Perandin F. Caldrer S. Formenti F. . (2021). Molecular techniques for the genomic viral RNA detection of West Nile, Dengue, Zika and Chikungunya arboviruses: a narrative review. Expert Rev. Mol. Diagn. 21, 591–612. doi: 10.1080/14737159.2021.1924059 33910444

[B93] Nguyen H. C. Park H. Shin H.-J. Nguyen N. M. Nguyen A. T. V. Trinh T.-T. T. . (2019). Fluorescent immunosorbent assay for Chikungunya virus detection. Intervirology 62, 145–155. doi: 10.1159/000502823 31533104

[B94] Novi V. T. Meher A. K. Abbas A. (2025). Visualization methods for loop mediated isothermal amplification (LAMP) assays. Analyst 150, 588–599. doi: 10.1039/D4AN01287A 39895350

[B95] Nunes J. A. L. de Sousa J. R. Smith V. C. Quaresma J. S. da Costa Vasconcelos P. F. Chiang J. O. (2023). Immunological impact of cytokines on the chikungunya virus pathophysiology: a literature narrative review. Rev. Med. Virol. 33:e2441. doi: 10.1002/rmv.2441 37021332

[B96] Nunes J. G. C. Nunes B. T. D. Shan C. Moraes A. F. Silva T. R. de Mendonça M. H. R. . (2021). Reporter virus neutralization test evaluation for dengue and Zika virus diagnosis in flavivirus endemic area. Pathogens 10:840. doi: 10.3390/pathogens10070840 34357990 PMC8308650

[B97] Ohst C. Saschenbrecker S. Stiba K. Steinhagen K. Probst C. Radzimski C. . (2018). Reliable serological testing for the diagnosis of emerging infectious diseases. Adv. Exp. Med. Biol. 1062, 19–43. doi: 10.1007/978-981-10-8727-1_3 29845523

[B98] Okabayashi T. Sasaki T. Masrinoul P. Chantawat N. Yoksan S. Nitatpattana N. . (2015). Detection of Chikungunya virus antigen by a novel rapid immunochromatographic test. J. Clin. Microbiol. 53, 382–388. doi: 10.1128/JCM.02033-14 25411170 PMC4298496

[B99] OrbegozoJ. PalermoP. M. WattsD. M. (2025). Serological evidence of flavivirus infection among mothers of newborns in El Paso, Texas. Vector Borne Zoonotic Dis. 25, 607–615. doi: 10.1177/1530366725136751840854730

[B100] Ou T. P. Sorn S. Nguon K. In S. Ken S. Ly S. . (2025). Viral kinetics during acute Chikungunya virus infection: insights into potential role of monoclonal antibodies in viral clearance and prophylaxis using mathematical modeling. J. Med. Virol. 97:e70391. doi: 10.1002/jmv.70391 40358000

[B101] Panmei K. Syed A. H. Okafor O. Mammen S. Abraham A. M. (2025). Performance evaluation of TaqMan^TM^ Arbovirus Triplex Kit (ZIKV/DENV/CHIKV) for detection and differentiation of dengue and Chikungunya viral RNA in serum samples of symptomatic patients. J. Virol. Methods 332:115072. doi: 10.1016/j.jviromet.2024.115072 39571938

[B102] Patel P. Abd El Wahed A. Faye O. Prüger P. Kaiser M. Thaloengsok S. . (2016). A field-deployable reverse transcription recombinase polymerase amplification assay for rapid detection of the Chikungunya virus. PLoS Negl. Trop. Dis. 10:e0004953. doi: 10.1371/journal.pntd.0004953 27685649 PMC5042537

[B103] Pereira S. S. Andreata-Santos R. Pereira L. R. Soares C. P. Félix A. C. de Andrade P. M. J. C. . (2021). NS1-based ELISA test efficiently detects dengue infections without cross-reactivity with Zika virus. Int. J. Infect. Dis. 112, 202–204. doi: 10.1016/j.ijid.2021.09.009 34555500

[B104] Perez L. J. Perez-Restrepo L. S. Ciuoderis K. Usuga J. Moreno I. Vargas V. . (2025). Emergence, persistence, and positive selection of yellow fever virus in Colombia. Front. Microbiol. 16:1548556. doi: 10.3389/fmicb.2025.1548556 40260085 PMC12009951

[B105] Pezzi L. Reusken C. B. Weaver S. C. Drexler J. F. Busch M. LaBeaud A. D. . (2019). GloPID-R report on Chikungunya, O'nyong-nyong and Mayaro virus, part I: biological diagnostics. Antiviral Res. 166, 66–81. doi: 10.1016/j.antiviral.2019.03.009 30905821

[B106] Plebani M. Nichols J. H. Luppa P. B. Greene D. Sciacovelli L. Shaw J. . (2025). Point-of-care testing: state-of-the art and perspectives. Clin. Chem. Lab. Med. 63, 35–51. doi: 10.1515/cclm-2024-0675 38880779

[B107] Polanía-Espinosa D. Castro-Trujillo S. Narváez C. F. (2025). Clinical, virological, and antibody profiles of overlapping dengue and chikungunya virus infections in children from southern Colombia. PLoS Negl. Trop. Dis. 19:e0013260. doi: 10.1371/journal.pntd.0013260 40920827 PMC12435647

[B108] Pollak N. M. Olsson M. Ahmed M. Tan J. Lim G. Setoh Y. X. . (2023a). Rapid diagnostic tests for the detection of the four dengue virus serotypes in clinically relevant matrices. Microbiol. Spectr. 11, e02796–22. doi: 10.1128/spectrum.02796-22 36682882 PMC9927141

[B109] Pollak N. M. Olsson M. Marsh G. A. Macdonald J. McMillan D. (2023b). Evaluation of three rapid low-resource molecular tests for Nipah virus. Front. Microbiol. 13:1101914. doi: 10.3389/fmicb.2022.1101914 36845977 PMC9949527

[B110] Power G. M. Vaughan A. M. Qiao L. Sanchez Clemente N. Pescarini J. M. Paixão E. S. . (2022). Socioeconomic risk markers of arthropod-borne virus (arbovirus) infections: a systematic literature review and meta-analysis. BMJ Glob. Health 7:e007735. doi: 10.1136/bmjgh-2021-007735 35428678 PMC9014035

[B111] Powers J. M. Lyski Z. L. Weber W. C. Denton M. Streblow M. M. Mayo A. T. . (2023). Infection with chikungunya virus confers heterotypic cross-neutralizing antibodies and memory B-cells against other arthritogenic alphaviruses predominantly through the B domain of the E2 glycoprotein. PLoS Negl. Trop. Dis. 17:e0011154. doi: 10.1371/journal.pntd.0011154 36913428 PMC10036167

[B112] Ramadona A. L. Tozan Y. Wallin J. Lazuardi L. Utarini A. Rocklöv J. (2023). Predicting the dengue cluster outbreak dynamics in Yogyakarta, Indonesia: a modelling study. Lancet Reg. Health Southeast Asia 15:100209. doi: 10.1016/j.lansea.2023.100209 37614350 PMC10442971

[B113] Ramírez A. L. Colmant A. M. G. Warrilow D. Huang B. Pyke A. T. McMahon J. L. . (2020). Metagenomic analysis of the virome of mosquito excreta. mSphere 5, e00587–20. doi: 10.1128/mSphere.00587-20 32907949 PMC7485684

[B114] Raulino R. Thaurignac G. Butel C. Villabona-Arenas C. J. Foe T. Loul S. . (2021). Multiplex detection of antibodies to Chikungunya, O'nyong-nyong, Zika, Dengue, West Nile and Usutu viruses in diverse non-human primate species from Cameroon and the Democratic Republic of Congo. PLoS Negl. Trop. Dis. 15:e0009028. doi: 10.1371/journal.pntd.0009028 33476338 PMC7853492

[B115] Ribeiro dos Santos G. Jawed F. Mukandavire C. Deol A. Scarponi D. Mboera L. E. G. . (2025). Global burden of chikungunya virus infections and the potential benefit of vaccination campaigns. Nat. Med. 31, 2342–2349. doi: 10.1038/s41591-025-03703-w 40495015 PMC12283390

[B116] Ribeiro M. R. C. Khouri R. Sousa P. S. Branco M. R. F. C. Batista R. F. L. Costa E. P. F. . (2020). Plaque Reduction Neutralization Test (PRNT) in the Congenital Zika Syndrome: positivity and associations with laboratory, clinical, and imaging characteristics. Viruses 12:1244. doi: 10.3390/v12111244 33142747 PMC7692785

[B117] Rodrigues A. M. Souza R. R. M. Fonseca L. M. D. S. Rolo C. D. A. Carvalho R. H. Sardi S. I. . (2020). Genomic surveillance of the Chikungunya Virus (CHIKV) in Northeast Brazil after the first outbreak in 2014. Rev. Soc. Bras. Med. Trop. 53:e20190583. doi: 10.1590/0037-8682-0583-2019 32578706 PMC7310362

[B118] Rutkowski N. Dong Y. Dimopoulos G. (2020). Field-deployable molecular diagnostic platform for arbovirus detection in Aedes aegypti. Parasit. Vectors 13:489. doi: 10.1186/s13071-020-04357-y 32972453 PMC7513541

[B119] Sajikumar S. Sureshkumar N. Sai V. V. R. Jyoti V. Sunil S. Srinivasan R. (2026). Development of a PCB-based electrochemical aptasensor for Chikungunya virus protein detection. Analyst 151, 1153–1162. doi: 10.1039/D5AN01261A 41568984

[B120] Sajith A. Iyengar V. Varamballi P. Mukhopadhyay C. Nittur S. (2025). Diagnostic utility of real-time RT-PCR for chikungunya virus detection in the acute phase of infection: a retrospective study. Ann. Med. 57:2523559. doi: 10.1080/07853890.2025.2523559 40570284 PMC12203700

[B121] Salgado B. B. de Jesus Maués F. C. Jordão M. Pereira R. L. Toledo-Teixeira D. A. Parise P. L. . (2023). Antibody cross-reactivity and evidence of susceptibility to emerging Flaviviruses in the dengue-endemic Brazilian Amazon. Int. J. Infect. Dis. 129, 142–151. doi: 10.1016/j.ijid.2023.01.033 36736575

[B122] Sawal H. A. Niazi S. K. Ghani E. Noor M. (2021). Phylogenetic and sequencing analysis of Chikungunya virus strains from local Pakistani population. J. Pak. Med. Assoc. 71, 2335–2339. doi: 10.47391/JPMA.01-166 34974566

[B123] Sekaran S. D. Ismail A. A. Thergarajan G. Chandramathi S. Rahman S. K. H. Mani R. R. . (2022). Host immune response against DENV and ZIKV infections. Front. Cell. Infect. Microbiol. 12:975222. doi: 10.3389/fcimb.2022.975222 36159640 PMC9492869

[B124] Sena B. F. Herrera B. B. Bruneska Gondim Martins D. Lima Filho J. L. (2024). Advancing arbovirus diagnosis in Brazil: strengthening diagnostic strategies and public health data collection. Braz. J. Infect. Dis. 28:103766. doi: 10.1016/j.bjid.2024.103766 38802065 PMC11153883

[B125] Sharma P. Hasan M. R. Naikoo U. M. Khatoon S. Pilloton R. Narang J. (2024). Aptamer based on silver nanoparticle-modified flexible carbon ink printed electrode for the electrochemical detection of chikungunya virus. Biosensors 14:344. doi: 10.3390/bios14070344 39056620 PMC11274990

[B126] Shete A. M. Jain R. Mohandas S. Pardeshi P. Yadav P. D. Gupta N. . (2022). Development of Nipah virus-specific IgM and IgG ELISA for screening human serum samples. Indian J. Med. Res. 156, 429–434. doi: 10.4103/ijmr.ijmr_2737_21 36510890 PMC10101364

[B127] Shurtleff A. C. Biggins J. E. Keeney A. E. Zumbrun E. E. Bloomfield H. A. Kuehne A. . (2012). Standardization of the filovirus plaque assay for use in preclinical studies. Viruses 4, 3511–3530. doi: 10.3390/v4123511 23223188 PMC3528277

[B128] Siew Z. Y. Seow I. Lim X. R. Tang C. Z. Djamil F. M. Ong G. K. . (2025). Arboviruses: the hidden danger of the tropics. Arch. Virol. 170:140. doi: 10.1007/s00705-025-06314-5 40418376

[B129] Siew Z. Y. Tang C. Z. Musa S. N. A. Seow I. Johari N. A. Leong P. P. . (2026). Treeshrews as a potential reservoir: first detection of dengue virus serotype 2 in Malaysian treeshrew faeces. J. Virol. Methods 339:115256. doi: 10.1016/j.jviromet.2025.115256 40897249

[B130] Simo F. B. N. Bigna J. J. Well E. A. Kenmoe S. Sado F. B. Y. Weaver S. C. . (2019). Chikungunya virus infection prevalence in Africa: a contemporaneous systematic review and meta-analysis. Public Health 166, 79–88. doi: 10.1016/j.puhe.2018.09.027 30468973

[B131] Sridhar S. Tonto P. B. Lumkong L. Netto E. M. Brites C. Wang W.-K. . (2024). RT-RPA as a dual tool for detection and phylogenetic analysis of epidemic arthritogenic alphaviruses. Sci. Rep. 14:30134. doi: 10.1038/s41598-024-81763-7 39627454 PMC11615341

[B132] Su W. Jiang L. Lu W. Xie H. Cao Y. Di B. . (2022). A serotype-specific and multiplex PCR method for whole-genome sequencing of dengue virus directly from clinical samples. Microbiol. Spectr. 10:e0121022. doi: 10.1128/spectrum.01210-22 36094197 PMC9602986

[B133] Takumi-Tanimukai Y. Yamamoto S. Ogasawara N. Nakabayashi S. Mizuta K. Yamamoto K. . (2022). A hydroxypropyl methylcellulose plaque assay for human respiratory syncytial virus. J. Virol. Methods 304:114528. doi: 10.1016/j.jviromet.2022.114528 35358640

[B134] Tang Z. Yamada H. Kraupa C. Canic S. Busquets N. Talavera S. . (2020). High sensitivity of one-step real-time reverse transcription quantitative PCR to detect low virus titers in large mosquito pools. Parasit. Vectors 13, 460. doi: 10.1186/s13071-020-04327-4 32907625 PMC7488135

[B135] Tchouassi D. P. Kisero R. O. Rotich G. Dunlap C. Torto B. Muturi E. J. (2024). Next generation sequencing improves the resolution of detecting mixed host blood meal sources in field collected arboviral mosquito vectors. Med. Vet. Entomol. 38, 407–415. doi: 10.1111/mve.12725 38747253

[B136] Tee K. K. Mu D. Xia X. (2025). Explosive chikungunya virus outbreak in China. Int. J. Infect. Dis. 161:108089. doi: 10.1016/j.ijid.2025.108089 41016657

[B137] Thannickal S. A. Battini L. Spector S. N. Noval M. G. Álvarez D. E. Stapleford K. A. (2024). Changes in the chikungunya virus E1 glycoprotein domain II and hinge influence E2 conformation, infectivity, and virus-receptor interactions. J. Virol. 98, e00679–24. doi: 10.1128/jvi.00679-24 38842335 PMC11265345

[B138] Thao T. T. N. de Bruin E. Phuong H. T. Thao Vy N. H. van den Ham H.-J. Wills B. A. . (2021). Using NS1 flavivirus protein microarray to infer past infecting dengue virus serotype and number of past dengue virus infections in Vietnamese individuals. J. Infect. Dis. 223, 2053–2061. doi: 10.1093/infdis/jiaa018 31967302 PMC8205622

[B139] Theillet G. Grard G. Galla M. Maisse C. Enguehard M. Cresson M. . (2019). Detection of chikungunya virus-specific IgM on laser-cut paper-based device using pseudo-particles as capture antigen. J. Med. Virol. 91, 899–910. doi: 10.1002/jmv.25420 30734316

[B140] Thompson K. Lyons S. Malone K. Fryk J. Pyke A. Murton K. (2026). Locally acquired dengue in Townsville, Australia, 2024-2025: an outbreak report in a non-endemic region with wMel Wolbachia-Infected Aedes aegypti. Trop. Med. Infect. Dis. 11:66. doi: 10.3390/tropicalmed11030066 41893402 PMC13029950

[B141] Torres do Couto M. T. Galdino da Silva Júnior A. Pereira Dos Santos Avelino K. Y. Vega Gonzales Gil L. H. Cordeiro M. T. Lima de Oliveira M. D. . (2024). Development of optical and electrochemical immunodevices for dengue virus detection. Anal. Methods 16, 3539–3550. doi: 10.1039/D4AY00514G 38780022

[B142] Tsai W.-Y. Driesse K. Tsai J.-J. Hsieh S.-C. Sznajder Granat R. Jenkins O. . (2020). Enzyme-linked immunosorbent assays using virus-like particles containing mutations of conserved residues on envelope protein can distinguish three flavivirus infections. Emerg. Microbes Infect. 9, 1722–1732. doi: 10.1080/22221751.2020.1797540 32684139 PMC7473235

[B143] Uhlikova N. Almeida M. I. G. S. McKelvie I. D. Kolev S. D. (2024). Microfluidic paper-based analytical device for the speciation of inorganic nitrogen species. Talanta 271:125671. doi: 10.1016/j.talanta.2024.125671 38306810

[B144] Umair M. Yasir M. Jamal Z. Hakim R. Saeed S. Y. Salman M. . (2025). Whole-Genome sequencing of Chikungunya Virus (CHIKV) from Pakistan: detection of the East/Central/South African (ECSA) genotype during the 2024 outbreak in Mansehra. PLoS ONE 20:e0329856. doi: 10.1371/journal.pone.0329856 40857268 PMC12380281

[B145] Umaña J. D. Montenegro-Torres J. Otero J. Tavera-Cifuentes M. C. Niño-Machado N. González-Uribe C. . (2026). Endemic channel parametrization in dengue surveillance: methodological assessment of retrospective windows, outbreak trends, and zero-case periods in Colombia. JMIR Public Health Surveill. 12:e79914. doi: 10.2196/79914 42102234 PMC13155432

[B146] Varghese J. De Silva I. Millar D. S. (2023). Latest advances in arbovirus diagnostics. Microorganisms 11:1159. doi: 10.3390/microorganisms11051159 37317133 PMC10223626

[B147] Verma J. Hasan A. Sunil S. Subbarao N. (2022). In silico identification and in vitro antiviral validation of potential inhibitors against Chikungunya virus. J. Comput. Aided Mol. Des. 36, 521–536. doi: 10.1007/s10822-022-00463-4 35789450

[B148] Wang M. Liu K. Guo D. Lv Y. Wang X. (2025). Arbovirus infections and epigenetic mechanisms; a potential therapeutic target. Rev. Med. Virol. 35:e70033. doi: 10.1002/rmv.70033 40155348

[B149] Wang M. Wang L. Leng P. Guo J. Zhou H. (2024). Drugs targeting structural and nonstructural proteins of the chikungunya virus: a review. Int. J. Biol. Macromol. 262:129949. doi: 10.1016/j.ijbiomac.2024.129949 38311132

[B150] Wang S. Liu Y. Wang Y. Zhou L. Liu J. (2026). Global and regional burden of chikungunya from 2004 to 2024: a worldwide observational study. J. Glob. Health 16:04055. doi: 10.7189/jogh.16.04055 41678821 PMC12902706

[B151] Wang S. M. Ali U. H. Sekaran S. D. Thayan R. (2016). Detection and quantification of chikungunya virus by real-time RT-PCR assay. Methods Mol. Biol. 1426, 105–117. doi: 10.1007/978-1-4939-3618-2_10 27233265

[B152] Waturangi D. E. (2024). Enumeration of bacteriophages by plaque assay. Methods Mol. Biol. 2738, 147–153. doi: 10.1007/978-1-0716-3549-0_9 37966597

[B153] Weber W. C. Streblow Z. J. Kreklywich C. N. Denton M. Sulgey G. Streblow M. M. . (2024). The Approved Live-Attenuated Chikungunya Virus Vaccine (IXCHIQ^®^) elicits cross-neutralizing antibody breadth extending to multiple arthritogenic alphaviruses similar to the antibody breadth following natural infection. Vaccines 12:893. doi: 10.3390/vaccines12080893 39204019 PMC11359099

[B154] Wesselmann K. M. Baronti C. Nougairède A. Thirion L. De Lamballerie X. Charrel R. . (2025). Development and evaluation of a duplex RT-qPCR assay for the detection and identification of Mayaro and chikungunya viruses. J. Clin. Microbiol. 63:e00420–25. doi: 10.1128/jcm.00420-25 40608324 PMC12345196

[B155] Wesselmann K. M. Luciani L. Thirion L. de Lamballerie X. Charrel R. Pezzi L. (2024). Analytical and clinical evaluation of a duplex RT-qPCR assay for the detection and identification of o'nyong-nyong and chikungunya virus. Emerg. Microbes Infect. 13:2429650. doi: 10.1080/22221751.2024.2429650 39530886 PMC11587735

[B156] Würsch D. Rojas-Montes O. Maldonado-Rodríguez A. Sevilla-Reyes E. Cevallos A. M. Sánchez-Burgos G. . (2023). Dried serum samples for antibody detection in arthropod-borne virus infections are an effective alternative to serum samples. Am. J. Trop. Med. Hyg. 109, 933–936. doi: 10.4269/ajtmh.22-0742 37640289 PMC10551088

[B157] Xiao P. Hao Y. Yuan Y. Ma W. Li Y. Zhang H. . (2025). Emerging West African genotype chikungunya virus in mosquito virome. Virulence 16:2444686. doi: 10.1080/21505594.2024.2444686 39715491 PMC11702961

[B158] Xu C. Chen Y. Zhu G. Wu H. Jiang Q. Zhang R. . (2024). A portable, integrated, sample-in result-out nucleic acid diagnostic device for rapid and sensitive chikungunya virus detection. Micromachines 15:663. doi: 10.3390/mi15050663 38793236 PMC11123350

[B159] Yang H. Patel D. J. (2024). Structures, mechanisms and applications of RNA-centric CRISPR-Cas13. Nat. Chem. Biol. 20, 673–688. doi: 10.1038/s41589-024-01593-6 38702571 PMC11375968

[B160] Yin B. Zhang Q. Xia X. Li C. Ho W. K. H. Yan J. . (2022). A CRISPR-Cas12a integrated SERS nanoplatform with chimeric DNA/RNA hairpin guide for ultrasensitive nucleic acid detection. Theranostics 12, 5914–5930. doi: 10.7150/thno.75816 35966585 PMC9373821

[B161] Zajac M. D. Ortega M. T. Moore S. M. (2023). Development and evaluation of an enzyme-linked immunosorbent assay targeting rabies-specific IgM and IgG in Human Sera. Viruses 15:874. doi: 10.3390/v15040874 37112853 PMC10142732

[B162] Zhang Y. Wu J. Cheng X. Yang Y. Wang X. Zhao X. . (2026). Global resurgence of Chikungunya virus: outbreak drivers and emerging solutions. Emerg. Microbes Infect. 15:2603714. doi: 10.1080/22221751.2025.2603714 41392880 PMC12777866

[B163] Zhuang S. Li J. Fang Z. Zhou H. Zhang R. He J. . (2026). Phosphorothioate modification-regulated one-pot CRISPR assay for arbovirus detection. ACS Infect. Dis. 12, 1423–1433. doi: 10.1021/acsinfecdis.6c00013 41867107

[B164] Zimmerman O. Zimmerman M. I. Raju S. Nelson C. A. Errico J. M. Madden E. A. . (2023). Vertebrate-class-specific binding modes of the alphavirus receptor MXRA8. Cell 186, 4818–4833.e25. doi: 10.1016/j.cell.2023.09.007 37804831 PMC10615782

